# NSD2 upregulation is driven by high-risk HPV E6/E7 and disrupts epithelial differentiation in HPV-associated head and neck cancer

**DOI:** 10.1186/s13046-025-03631-0

**Published:** 2026-01-08

**Authors:** Lavinia Ghiani, Simona Citro, Alessandro Medda, Mirko Doni, Farkhondeh Ghoryani, Roberta Noberini, Ottavio Croci, Fausto Maffini, Claudia Miccolo, Laura Monteleone, Marta Tagliabue, Rita De Berardinis, Stefano Campaner, Tiziana Bonaldi, Mohssen Ansarin, Susanna Chiocca

**Affiliations:** 1 Department of Experimental Oncology, IEO, European Institute of Oncology IRCCS, via Adamello 16, Milan, 20139 Italy; 2 Center for Genomic Science of IIT, CGS@SEMM (Istituto Italiano di Tecnologia at European School of Molecular Medicine), Fondazione Istituto Italiano di Tecnologia (IIT), Milan, Italy; 3 Department of Surgical Pathology, European Institute of Oncology IRCCS, Milan, 20141 Italy; 4 Department of Otorhinolaryngology and Head and Neck Surgery, European Institute of Oncology, IRCCS, Via Ripamonti 435, Milan, 20141 Italy; 5 Department of Molecular Medicine, University of Padua, Padua, Italy; 6 Department of Oncology and Haemato-Oncology (DIPO), University of Milano, Via Santa Sofia 9/1, Milano, 20121 Italy

**Keywords:** Head and neck squamous cell carcinoma (HNSCC), Head and neck cancer (HNC), Human papillomavirus (HPV), Epigenetics, Histone post-translational modifications (hPTMs), NSD2, Epithelial cell differentiation.

## Abstract

**Background:**

Head and Neck Squamous Cell Carcinoma (HNSCC) are classified in two main subtypes: HPV-positive (HPV+), driven by human papillomavirus (HPV) infections, and HPV-negative (HPV-), associated with environmental risk factors. Despite molecular and clinicopathological differences, neither subtype has effective tailored therapies. Since high-risk HPV oncoproteins E6/E7 affect several epigenetic regulators, characterizing the epigenetic landscape of HPV+ and HPV- HNSCC may uncover novel subtype-specific biomarkers and therapeutic targets.

**Methods:**

Histone post-translational modifications were profiled in HPV+ and HPV- HNSCC tissues and cell lines using super-SILAC mass spectrometry. The same analysis was performed and combined with RNA-sequencing on E6/E7-transduced human primary keratinocytes (HKs) to identify relevant histone modifiers affected by HPV oncoproteins. Candidate gene was validated via E6/E7-mediated-siRNA knockdown in HPV + cell lines. Western Blot, RT-qPCR and Immunohistochemistry assessed gene expression. NSD2 expression was examined in patients’ tissue samples, TCGA data and 14 HNSCC cell lines. shRNA-mediated NSD2 knockdown followed by RNA-seq, cell proliferation and migration assays evaluated its oncogenic role in HNSCC. CaCl_2_ treatments were used to investigate NSD2’s role in epithelial differentiation, while ALDH-positive cells were quantified by flow-cytometry. NSD2 overexpression was used to confirm results.

**Results:**

HPV+ HNSCC exhibited elevated H3K36me2 levels, compared to HPV-. This alteration is driven by E6/E7-induced NSD2 upregulation. NSD2, a histone methyltransferase specific for H3K36 di-methylation is overexpressed in HPV+ relative to HPV- HNSCC and in both subtypes compared to normal tissue, suggesting crucial implications in HNSCC. Functional assays revealed that NSD2 promotes cell proliferation and migration in both the subtypes. Notably, we identified a novel role for NSD2 in inhibiting epithelial cell differentiation, particularly in HPV+ HNSCC, where its upregulation mediates the E6/E7-induced differentiation blockade.

**Conclusions:**

We identified a novel HPV-driven epigenetic signature in HNSCC marked by increased H3K36me2 and its writer, NSD2. Our study highlights H3K36me2 as a potential biomarker for patient stratification and positions NSD2 as a promising therapeutic target across HNSCC subtypes, modulating both common and subtype-specific oncogenic pathways. Specifically, NSD2 inhibition in HPV+ tumors restores epithelial differentiation, offering a potential strategy to arrest tumor progression.

**Supplementary Information:**

The online version contains supplementary material available at 10.1186/s13046-025-03631-0.

## Background

Head and Neck Squamous Cell Carcinoma (HNSCC) accounts for 90% of Head and Neck Cancers (HNC) and is the seventh most common cancer worldwide, with nearly 890,000 new cases and 450,000 deaths reported in 2020 [1, 2]. HNSCC arises from the epithelial cells of the mucosal linings of various anatomical regions, including the nasopharynx, oral cavity, oropharynx, hypopharynx, and larynx [3, 4]. The primary risk factors for HNSCC include alcohol and tobacco consumption, poor oral hygiene and persistent high-risk Human Papillomavirus (HPV) infections. Based on HPV status, HNSCC can be classified into two distinct subtypes: the HPV-negative (HPV−) and the HPV-positive (HPV+) [3, 4]. HPV+ HNSCC predominantly occur in the oropharynx, with 40–90% of oropharyngeal squamous cell carcinomas (OPSCC) being HPV-associated [4, 5]. Despite the well-established molecular and clinical differences between HPV+ and HPV− HNSCC —recognized in the AJCC 8^th^ edition staging system— current treatment approaches remain largely uniform, relying mainly on surgery, radiotherapy, and chemotherapy, and the overall survival and prognosis of patients remain poor [3, 4, 6, 7]. This underscores the urgent need to comprehensive characterize the molecular mechanisms distinguishing these subtypes, with the goal of identifying novel clinical biomarkers and developing more targeted therapeutic approaches.

HPVs are double-stranded DNA viruses, classified as either low-risk (lr) or high-risk (hr), with hr-HPVs, as HPV16 and 18, being strongly associated with cancer development. Their oncogenic potential is mainly driven by the E6 and E7 oncoviral proteins, which alter many cellular pathways and lead the key tumor suppressors p53 and pRb to proteasomal degradation [3, 8, 9]. Among the others, E6/E7 impair epithelial differentiation and increase cell plasticity, thus disrupting the proper balance between cell proliferation and cell differentiation which is crucial for maintaining tissue homeostasis and preventing malignant transformation [3, 10, 11]. As such, differentiation-based therapies, which aim at promoting terminal differentiation and depleting the tumor’s proliferative compartment, are emerging as promising strategies in cancer treatment [11–13]. Notably, E6 and E7 also target epigenetic regulators of the host cell [14–16]. Epigenetic alterations, including changes in histone post-translational modifications (hPTMs), play a pivotal role in oncogenesis and their reversible nature makes them attractive targets for therapeutic intervention. Reprogramming the epigenetic landscape of tumoral cells may restore a non-mitotic, terminally differentiated state, thereby reducing tumorigenic potential [17]. Several epigenetic drugs (epi-drugs) are currently in clinical trials, either as monotherapies or in combination with other agents, with some already approved for cancer treatment [18].

Aberrant expression of histone-modifying enzymes and altered hPTM patterns have been reported in HNSCC, with growing evidence pointing to epigenetic differences between HPV+ and HPV− tumors [16, 19]. While DNA methylation has been extensively studied in HNSCC, revealing key differences between HPV+ and HPV- subtypes [12, 13, 16, 51], a comprehensive analysis of hPTMs in HNSCC remains lacking. Addressing this gap could lead to identifying novel biomarkers for patient stratification and new epigenetic targets for therapeutic intervention.

NSD2, also known as MMSET or WHSC1, is a histone methyltransferase responsible for mono- and di-methylation of histone H3 on lysine 36 (H3K36me2). Its overexpression or hyperactivation, and the associated global increases of H3K36me2 levels have been linked to various cancers, including HNSCC [20–22]. Although limited studies have explored the role of NSD2 in HNSCC, evidence suggests that its overexpression contributes to increase cell proliferation and promote epithelial-to-mesenchymal transition (EMT) [22–26]. However, potential differences in NSD2 role between HPV+ and HPV− HNSCC have not been addressed, despite indications of a context-dependent role for NSD2 [27, 28].

In this study, we quantitatively profiled the hPTM landscape of HPV+ and HPV− HNSCC. Our results revealed significantly elevated levels of H3K36me2 in HPV+ HNSCC compared to HPV− HNSCC. Importantly, we observed, for the first time, that hr-HPV E6/E7 oncoproteins upregulate NSD2 and drive H3K36me2 enrichment. Additionally, we observed higher levels of NSD2 in HPV+ compared to HPV- HNSCC. In this context, we observed that NSD2 upregulation not only promotes cell proliferation and EMT in both the subtypes, but also impairs cell differentiation mostly within the HPV+ subtype. This is consistent with the known functions of both NSD2 and H3K36me2 in controlling cell plasticity, a key feature of cancer initiation and progression [22, 26, 29–35].

## Materials and methods

### Cell lines

Head and Neck cell lines were obtained from different sources. The UM-SCC-4, UM-SCC-6, UM-SCC-10A, UM-SCC-18, UM-SCC-19, UM-SCC-23, UM-SCC-28 and UM-SCC-47 cell lines were generated by Prof. Thomas E. Carey. The UD-SCC-2 cell line was kindly provided by Prof. Henning Bier. The 93-VU-147T cell line was kindly provided by Dr. Martin Rooimans, University Medical Centre, Amsterdam, the Netherland. The UM-SCC-17A, UM-SCC-17B, UM-SCC-104 cell lines were purchased by Merck S.p.A. The UPCI:SCC-154, UPCI:SCC-152, UPCI:SCC-90 cell lines were acquired from ATCC^®^. Characteristics of HNC cell lines, such as HPV-positivity, subsite of origin and sex of the donor are mainly summarized in [36, 37], while for the others we report here some information: UM-SCC-17A is a HPV- cell lines derived from the larynx of a female donor, UM-SCC-17B is derived from metastasis of the same donor [38]; UPCI:SCC-152 is a HPV+ cell line derived from the hypopharynx of a male donor and is a recurrence of UPCI:SCC-90 [37].

All the cell lines were grown in Dulbecco’s modified Eagle’s medium (DMEM) with stable glutamine and sodium pyruvate (EuroClone) and supplemented with 10% fetal bovine serum (FBS) of North America origin (Euroclone), non-essential amino acids (Lonza), 250 µM/ml penicillin and 25 µM/ml streptomycin (Euroclone).

HEK293T and Phoenix-Ampho packaging cells were cultured in DMEM with stable glutamine and sodium pyruvate (EuroClone), supplemented with 10% FBS (South America origin) (Euroclone), 250 µM/ml penicillin and 25 µM/ml streptomycin (Euroclone). All cells were maintained at 37 °C in a 5% CO_2_ buffered incubator.

Every 6 months all cell lines were authenticated by short tandem repeat (STR) profiling and tested for mycoplasma contamination.

### Human primary keratinocytes

Skin tissues were collected from male and female donors and anonymized via standardized operative procedures approved by the European Institute of Oncology (IEO) Ethical Board. Informed consent was obtained from all patients. Adult human epidermal keratinocytes (HKs) were derived according to previously described protocol [39]. The isolated primary keratinocytes were cultured in Keratinocyte Serum-Free Medium (KSFM; Gibco), supplemented with bovine pituitary extract (BPE, 30 ug/mL; Gibco) and epidermal growth factor (EGF, 0,2 ng/mL; Gibco). HKs from passages 1 to 6 were used for the experiments. HKs were maintained at 37 °C in a 5% CO_2_ buffered incubator.

### Patients’ samples

Tumoral and adjacent normal tissue samples were separately collected, and preserved according to the appropriate procedures, from both male and female HNSCC patients upon surgery at the European Institute of Oncology, IRCCS (IEO) (Milan), through the Biobank for Translational Medicine Unit (B4MED). We both received Formalin-Fixed Paraffin-Embedded (FPPE) and cryopreserved tissue samples. For each sample we received all clinical information, such as HPV status, sex, anatomic site and subsite of, histological grade (G1, G2, G3), tumor stage, etc. FFPE tissue samples were processed for mass spectrometry analyses and immunohistochemistry. To determine HPV positivity in oropharyngeal cancers, p16INK4 IHC was used. According to established guidelines, a carcinoma was considered HPV-associated, if staining was observed in more than 75% of neoplastic cells. In oropharyngeal cancer samples, were p16INK4 IHC staining was not unambiguous, the diagnostic test INNO-LiPA HPV Genotyping Extra II Amp (Fujirebio Europe N.V) was used to identify HPV by PCR targeting a 65-bp region of L1 gene. In our study, HPV-PCR was performed in 4 samples out of 32. Cryopreserved tissues were snap-frozen in liquid nitrogen and preserved at −80 °C.

### Transductions, transfections and plasmids (siRNA sequences, shRNA sequences)

Selected HNSCC cell lines were seeded in 60 mm plates and transfected with Lipofectamine 3000 (Thermo Scientific #100022052) with siLuc or E6/E7 siRNAs following manufacturers’ instruction. siRNAs sequences were listed in Supplementary Table 1. 72 h after transfection, cells were collected for RT-qPCR and Western Blot analysis.

For retroviral and lentiviral transduction, 8 µg of the plasmids of interest were transfected respectively into Phoenix-Ampho or   HEK293T cell lines through the calcium-phosphate method. For lentiviral production cells were also transfected with the helper plasmids pCMV-VSV-G (3 µg) and pCMV-dR8.9 (6 µg). Eight hours post transfection the medium was changed, and 48 h post transfection, viral supernatant was collected, filtered through a 0,45 μm syringe-filter and added to target cells plates. Two cycles of infection of 3 h at 37 °C were performed for each plate supplemented with 8 µg/ml Polybrene (Merck). The following day, cells were splitted and selected with G-418 Sulfate (Gibco) for 5 days (700 µg/ml for UM-SCC-4 and UM-SCC-18; 600 µg/ml for 93-VU147-T; 1200 µg/ml for UM-SCC-47; 100 µg/ml for HKs) or with Puromycin and for 3 days (1 µg/ml for HNSCC cell lines; 0,2 µg/ml for HKs), according to the vector.

Retroviral plasmid pLXSN-6 E6/E7, pLXSN-10 E6/E7, pLXSN-16 E6/E7, pLXSN-18 E6/E7 were generated as described in [39].

Lentiviral plasmid encoding shNSD2_A has been purchased from Sigma Mission (product code: TRCN0000274182) and shNSD2_B has been manually designed. Oligos sequences were listed in Supplementary Table 2. Oligos were subcloned as described in [40] into the pLKO.1 lentiviral vector containing puromycin resistance (Sigma-Aldrich). As a control, a scrambled sequence was used and subcloned into the pLKO.1 vector.

The pLVXN-Neo-NSD2 lentiviral vector (expressing human wild-type NSD2) was purchased from Addgene (Plasmid #86010).

### LC/MS analysis of histone PTMs

Histones were purified from 6–7*10^6^ HNSCC cells as described [41]. For formalin-fixed paraffin-embedded (FFPE) tissues, histones were enriched from manually macrodissected tumoral areas from 6 to 8 5 μm-thick tissue sections, following the pathology tissue analysis of histones by mass spectrometry (PAT-H-MS) protocol [41]. For super-SILAC (Stable-Isotope Labeling using Amino acids in Cell culture) Mass Spectrometry, approximately 5 µg of histone octamer were mixed with an equal amount of heavy-isotope labelled histones, which were used as an internal standard, and separated on a 17% SDS-PAGE gel. A mixture of labelled histones obtained from breast cancer cell lines were used as internal standard for the analysis of HNSCC cell lines [42], while labelled histones from different cancer cells were used for the analysis of FFPE samples and human keratinocytes [43]. Histone bands were excised, chemically acylated with propionic anhydride (FFPE samples and keratinocytes) or deuterated acetic anhydride (cell lines) and in-gel digested with trypsin. Propionic anhydride-derivatized peptides were further derivatized with phenyl isocyanate [44]. Peptide mixtures were separated by reversed-phase chromatography on an EASY-Spray column (Thermo Fisher Scientific), 25-cm long (inner diameter 75 μm, PepMap C18, 2 μm particles), which was connected online to a Q Exactive Plus instrument (Thermo Fisher Scientific) through an EASY-Spray™ Ion Source (Thermo Fisher Scientific), as described [44]. The acquired RAW data were analyzed using EpiProfile 2.0 [45], followed by manual validation. For each histone modified peptide, a % relative abundance (%RA) value for the sample (light channel - L) or the internal standard (heavy channel - H) was estimated. Light/Heavy (L/H) ratios of %RAs were then calculated and are reported in Supplementary Data. The removeBatchEffect function from the limma package [46] was used to correct the log2 L/H ratios for batch effects. The mass spectrometry data have been deposited to the ProteomeXchange Consortium [47] via the PRIDE partner repository with the dataset identifier PXD064152.

### CaCl_2_ differentiation inducing method

HKs were induced to differentiate by supplementing the medium with 1.2 mM CaCl₂. After 3 days of treatment cells were harvested and/or imaged. Phase-contrast pictures were acquired through the EVOS fl. microscope (Advanced Microscopy Group, Inc).

### RNA extraction, reverse transcription and qRT-PCR

Total RNA was extracted from cells with the Quick-RNA MiniPrep kit (Zymo Research ##D4020), following the manufacturer’s instruction). Cryopreserved tissues samples were instead processed for RNA extraction using AllPrep DNA/RNA/protein kit (Qiagen) following manufacturers’ instructions.

cDNA was generated by reverse transcription-PCR with with the LunaScript™ RT SuperMix Kit (New England Biolabs #E3010L). Relative levels of specific mRNAs were determined with Luna Universal qPCR master mix (New England Biolabs #M3003E) using the ViiA™ 7 Real-Time PCR System (Applied Biosystem) or the QuantStudio™ 6 Pro Real-Time PCR System (Applied Biosystem). The RPLP0 (Neutral Ribosomal Phosphoprotein P0) gene was used as a housekeeper gene for normalization and the expression of the indicated mRNAs was calculated using the 2^−ΔCT^ or 2^−ΔΔCT^ methods. Primers used are listed in Supplementary Table 3.

### Library preparation and RNA sequencing

Total RNA was extracted using Zymo Quick-RNA Kit (Zymo Research #D4020) from 4 biological replicates of HKs transduced with the empty or HPV-16 E6/E7- encoding pLXSN vector and from HNSCC cell lines (UM-SCC-4, UM-SCC-6, UM-SCC-18, UM-SCC-19, UD-SCC-2, UM-SCC-104, UPCI:SCC-152) transduced with scrambled or shNSD2_A plko.1 vectors. RNA quality and integrity were assessed with an Agilent 2100 bioanalyzer using an RNA 600 Nano kit (Agilent Technologies). 500 ng of total RNA were used to prepare library for RNA sequencing using the Truseq RNA Sample Prep Kit V2set B (Illumina #RS-122–2002) according to the manufacturer instructions. Sequencing was performed using NovaSeq 6000 system (Illumina) with a read length of 50 bp (paired-end).

### RNA-Seq bioinformatic analysis

Reads from HNSCC cell lines RNA-sequencing were processed using HTS-flow framework described in [48]. Reads were filtered using fastq masker (with options -Q33 -q 20 -r -N -v -i) and aligned to human genome hg38 with tophat software v. 2.0.8 with the parameters: -r 170 -p 8 --no-novel-juncs --no-novel-indels --librarytype fr-unstranded [49]. Differential gene expression analyses were performed using DESeq2 R package [50], considering the cell line as confounding factor. Genes were identified as DEGs when the following criteria were met: p-adjusted ≤ 0.05 and | log2fold change | ≥ 0.5. RNA-seq data from Human Primary keratinocytes transduced with E6/E7 were downloaded from GSE235662 and processed as in [40].

The heatmap showing the log2 fold change of genes in HPV- and HPV+ subtypes was built with custom R scripts considering significantly deregulated genes (padj ≤0.05) in at least one of the two subtypes (HPV- and HPV+) and applying a k-means clustering with k = 9.

Gene Ontology (GO) analyses were carried out using custom R scripts. Hypergeometric test was applied to determine the statistical significance of the gene signatures. C2, C5 and Hallmark gene sets of Molecular Signature Database (MSigDB) were used as databases (http://software.broadinstitute.org/). Data visualization of GO was carried out through GraphPad Prism version 8.00 software.

To obtain enrichment plots, gene set enrichment analyses were carried out using GSEA desktop application [51]. The analysis was performed using the following parameters: number of permutations = 1000, no collapsed, max gene set size = 500, min gene set size = 15. Genesets C2 all, C5, C6 and hallmarks from MSigDB were used for the analyses. A gene set was considered statistically significant if padj value was ≤ 0.05. Venn diagrams were built using the Venny interactive tool (https://bioinfogp.cnb.csic.es/tools/venny/index.html).

### Immunoblotting and antibodies

Upon harvesting, cells were lysed in a sodium dodecyl sulfate (SDS) lysis buffer made of 1:3 mix of buffer I (5% SDS, 0.15 M Tris-HCl [pH 6.8], 30% glycerol) and buffer II (25 mM Tris-HCl [pH 8.3], 50 mM NaCl, 0.5% NP-40, 0.1% SDS, 1 mM EDTA). Protease inhibitors were freshly added: 1 µg/mL leupeptin, 1 µg/mL aprotinin, 100 µg/mL PMSF, 0.5 mM NaF and 2 mM sodium Na_3_VO_4_.

Lysates were then sonicated and clarified by centrifugation and an equal amount of proteins for each sample was resuspended in denaturating sample loading buffer and boiled. Samples were loaded and resolved on SDS-polyacrylamide gel, blotted on a methanol-activated PVDF membrane (Immobilion-P #IPVH00010) and probed with the indicated antibodies: anti-NSD2 (ab75359,1:1000), anti-p63 (ab735, 1:1000), anti-IVL (sc-21748, 1:500), anti-VIM (ab8069, 1:1000), p53 (sc-126, 1:1000), E7 (sc-6981, 1:500), H3K36me2 (ab9049, 1:1000), H3 (sc-517576, 1:1000), Anti-Vinc (V9131, 1:10000), anti-β-actin (sc-47778, 1:2000), GAPDH (Ab8245, 1:5000), H4 total (Ab7311). Membranes were then incubated with the appropriate horseradish peroxidase (HRP) secondary antibodies (anti-mouse or anti-rabbit − 1:10000) and the signal was developed with ECL (Biorad Clarity #170–5061) and acquired with Chemidoc (Bio-Rad) or with Chemi iBright (Thermofisher). Densitometric analysis of the intensity of the protein bands was performed using ImageLab software (Bio-Rad).

### Immunohistochemistry

Human HNSCC FFPE sections, provided by the IEO hospital, were deparaffinized in histolemon and rehydrated. Antigen unmasking was performed for 30’ at 99 °C using or Citrate Buffer pH 6 (Unmasking Citrate Buffer #HK086-9 K). Only for NSD2 stainings, FFPE sections were deparaffinized with xylene and unmasked with EDTA pH 9 (Unmasking EDTA pH9 #S2367).

Slides were incubated for 5’ at RT with 3% H202 to quench endogenous peroxidases. After blocking in 4% BSA in TBS-T (Tween20 0,05%), samples were incubated overnight at 4 °C with the following antibodies: anti-NSD2 (ab75359, 6.6 µg/mL), anti-p63 (ab735, 10 µg/mL), anti-IVL (sc-21748, 1 µg/mL), anti-TGase1 (sc-166467, 2 µg/mL), anti-Ki67 (MA5-14520, 0.155 µg/mL). Incubation with secondary antibody ready to use (DAKO Envision system HRP rabbit or mouse) was performed for 30’ @ RT. Signal was revealed through incubation in peroxidase substrate solution (DAB DAKO) for 2’ to 10’ and nuclei were counterstained with hematoxylin. Tissue samples were dehydrated and mounted with the Eukit mounting medium (Bio-Optica 09–00250). Images were acquired using the Hamamatsu NanoZoomer S60 Microscope.

### Quantification of DAB nuclear signal from tissues

To measure the DAB signal intensity in tissues, the image analysis software QuPath version 0.5.0 was used [52]. As the marker is expressed in cell nuclei, the DAB signal was used to segment nuclei using the QuPath extention for StarDist algorithm [53]. Prior to nuclei detection, a multi-step preprocessing pipeline was applied to enhance segmentation accuracy, namely [1] color deconvolution to separate histological stains (DAB and hematoxylin); [2] hematoxylin and DAB staining were extracted and summed to create a composite image emphasizing nuclear morphology; [3] a median filter with a 2-pixel kernel was applied to reduce image noise while preserving nuclear boundaries; [4] percentile-based normalization was performed using the 20th and 80th percentiles to standardize image contrast across different samples and reduce batch effects. For each detected nucleus, comprehensive morphological and intensity measurements were automatically extracted, including shape parameters (area, circularity, eccentricity) and intensity features (mean, standard deviation pixel intensities across all image channels). Then, the mean intensities of all cells inside samples were calculated.

### Immunofluorescence

Cells were plated on glass coverslips and, when reached the appropriate density, were fixed with 4% paraformaldehyde for 15’and permeabilized by 10’incubation in Triton 0,1%. After 1 h of blocking in 2% BSA, cells were incubated overnight at 4 °C with the anti-NSD2 (ab75359, 10 µg/mL) primary antibodies, followed by incubation with a secondary anti-mouse AlexaFluor488 antibody (1:100, Jackson ImmunoResearch #715-545−150). To visualize F-actin, cells were incubated with TRIC-conjugated Phalloidin (1:50, Sigma-Aldrich #P1951) for 1 h at room temperature. Nuclei were stained with 4′,6-diamidino-2-phenylindole (DAPI, Merck #D9542). Glass coverslips were mounted on glass slides using 5 µL of glycerol (Sigma-Aldrich Merck) as mounting medium. Images were acquired with a 40x objective, using the Nikon spinning disk CSU-W1confocal microscopy and the Photometrics Prime BSI camera.

### Quantitative analysis of cell size

To calculate the area occupied by cells, a custom Python-based image segmentation pipeline exploiting the deep learning StarDist algorithm [53–55] to segment nuclei from the DAPI channel, and Cellpose algorithm [56] to segment whole cells from the Phalloidin channel were employed. After cell segmentation, the mean cell area was calculated.

### Cell proliferation assay

After selection, cells were plated for cell proliferation assays. Cells were plated in duplicate into 6-well plates at the appropriate density and cell proliferation was assessed at 3, 6 and 9 days after plating (T0), by counting cells with the TC20™ Automated Cell Counter (Biorad), upon trypan blue staining. For CellTiter-Glo^®^ Luminescent Cell Viability Assay (Promega), cells were plated in 96-well plates at the appropriate density and cell proliferation was assessed following the manufacturer’s instructions.

### Colony formation assay

After selection cells were plates in 6-well plates. 250 cells/well were plated for UM-SCC-19; l000 cells/well for UM-SCC-4, UM-SCC-6, UM-SCC-18, 93-VU147-T, UM-SCC-47; 2000 cells/well for UM-SCC-104 and UPCI:SCC-152. After 15–20 days from the plating (according to the cell line), cells were fixed and stained with a cristal violet solution. Images of the plates were acquired with the iBright Imaging System (Thermofisher Scientific) and an ImageJ (Fiji) tool was used to quantify the number of colonies of each well.

### Migration assays

Wound-Healing Assay and Transwell Migration Assay were used to evaluate the migrating phenotype. For wound-healing assay, cells were plated in triplicate in 12-well plates and when they reached a confluency of 90–95% the medium was changed to 0,5% FBS in order to reduce the confounding effect due to differences in proliferation rates between the control and the NSD2-silenced or -overexpressing cells. After approximately 16 h of serum-starvation, cell monolayer was wounded with a sterile 20 µl pipette tip. Cells were washed with PBS and rinsed with fresh medium maintaining the serum-starvation condition until the end of the experiment. Pictures were taken by brightfield and phase contrast microscopy (Evos fl., Advanced Microscopy Group, Inc) at two time points after wounding: at 0 h and at 4–48 h, according to the cell line.

Transwell Migration assay was performed using 24-well transwell chambers with 0,8 μm pores membranes (Corning #353097). Cells were trypsinized and counted: 100.000 cells resuspended in a volume of 200 µl of 0,5% FBS medium were seeded into the top-chamber. The outer chamber was filled with 800 µl of 20% FBS medium. According to the cell line, after 24 (UM-SCC-4, UM-SCC-19, 93-VU147-T) or 48 (UD-SCC-2) hours at 37 °C, cells were fixed and stained with cristal violet (Sigma-Aldrich #V5265) for 15 min at room temperature. The cells attached to the upper chamber were removed with cotton swabs. Pictures were acquired through brightfield and phase contrast microscopy (Evos fl., Advaanced Microscopy Group, Inc).

### Aldefluor assay

Aldefluor Assay was performed following the manufacturer instructions (ALDEFLUOR™ Kit, Stemcell™ Technologies). In detail, two days after plating, sub-confluent cells were harvested by trypsinization. 1 × 10^6^/mL or 500.000 cells were used for the experiment according to the cell line (1 × 10^6^/mL for UM-SCC-6, UM-SCC-18 and UPCI:SCC-152; 500.000 for UM-SCC-19) and stained with the ALDEFLUOR reagent. The sample was then splitted into “test”, in which ALDH reaction was assessed by fluorochrome positivity, and negative control “DEAB”, in which ALDH reaction was quenched, and thus, referred to as negative control. Cells were acquired with a BD FACS Celesta using the FacsDIVA Software (BD Bioscience). Analyses were performed using FlowJo 10.

### Sphere formation assay

To assess sphere forming capacity cells were trypsinized and counted. 2000 cells/well for UM-SCC-6, 93-VU147-T and UPCI: SCC-152 and 5000 cells/well for UM-SCC-19 were plated in 24-well ultra-low attachment plates in sphere media (DMEM-F12), 20 ng/ml EGF, 20 ng/ml b-FGF, B27 (Gibco, 17504-044), 1% methylcellulose). Fresh medium was added every 7days. The number of spheres formed per well was counted after 15–20 days according to the cell line. Only spheres with a diameter greater than 70 μm where considered for UM-SCC-6, UM-SCC-19 and UPCI:SCC-152 and greater than 50 μm for 93-VU147-T were considered.

### TCGA data analysis

mRNA expression levels for the genes of interest, expressed as Normalized RNA-seq Reads (log2(fpkm-uq + 1), were extracted from the TCGA PanCancer Atlas through the cBioPortal and the GDC data portal [57, 58] (https://portal.gdc.cancer.gov/). Patient samples with known HPV status and available RNA-seq data were grouped as HPV+ and HPV−, resulting in 72 HPV+ and 415 HPV- samples. Samples were also furtherly grouped according to their histological grade, resulting in 58 G1, 291 G2, 116 G3, 4 G4 samples. RNA expression data relative to normal adjacent tissues were available for 44 samples and extracted from the GDC data portal. Graphs and statistical analyses were built and performed using GraphPad Prism v10.0 (Graphpad Software, Inc., San Diego, California, USA).

### Statistical analysis

Statistical analysis was carried out using GraphPad Prism v.10 (GraphPad Software). Data are expressed as means ± SD of the indicated number of biological independent replicates. To compare two sample groups, either the *Student’s t-test* or the *Mann–Whitney U-test* was used depending on normality. Normality was assessed using *Shapiro Wilk’s normality test*. *Unpaired t-test* was used to compare treated/control groups when data from independent experiments were normalized relative to each control group average. *One-sample t-test* was performed when independent replicates were first normalized to their corresponding controls and then pooled together. ANOVA was used to compare more than two groups. *Spearman’s or Pearson’s* rank correlation coefficient were used to test correlation between variables. Details on statistical test are indicated in figure legends. Values of *p* ≤ 0.05 were considered significant. *,*P* ≤0.05; **,*P* ≤ 0.01; ***,≤0.001; ****, ≤0.0001; ns, not significant.

## Results

### HPV+ HNSCC exhibit higher H3K36me2 levels compared to HPV- HNSCC samples due to HPV16 E6/E7 oncoproteins

**Fig. 1:**
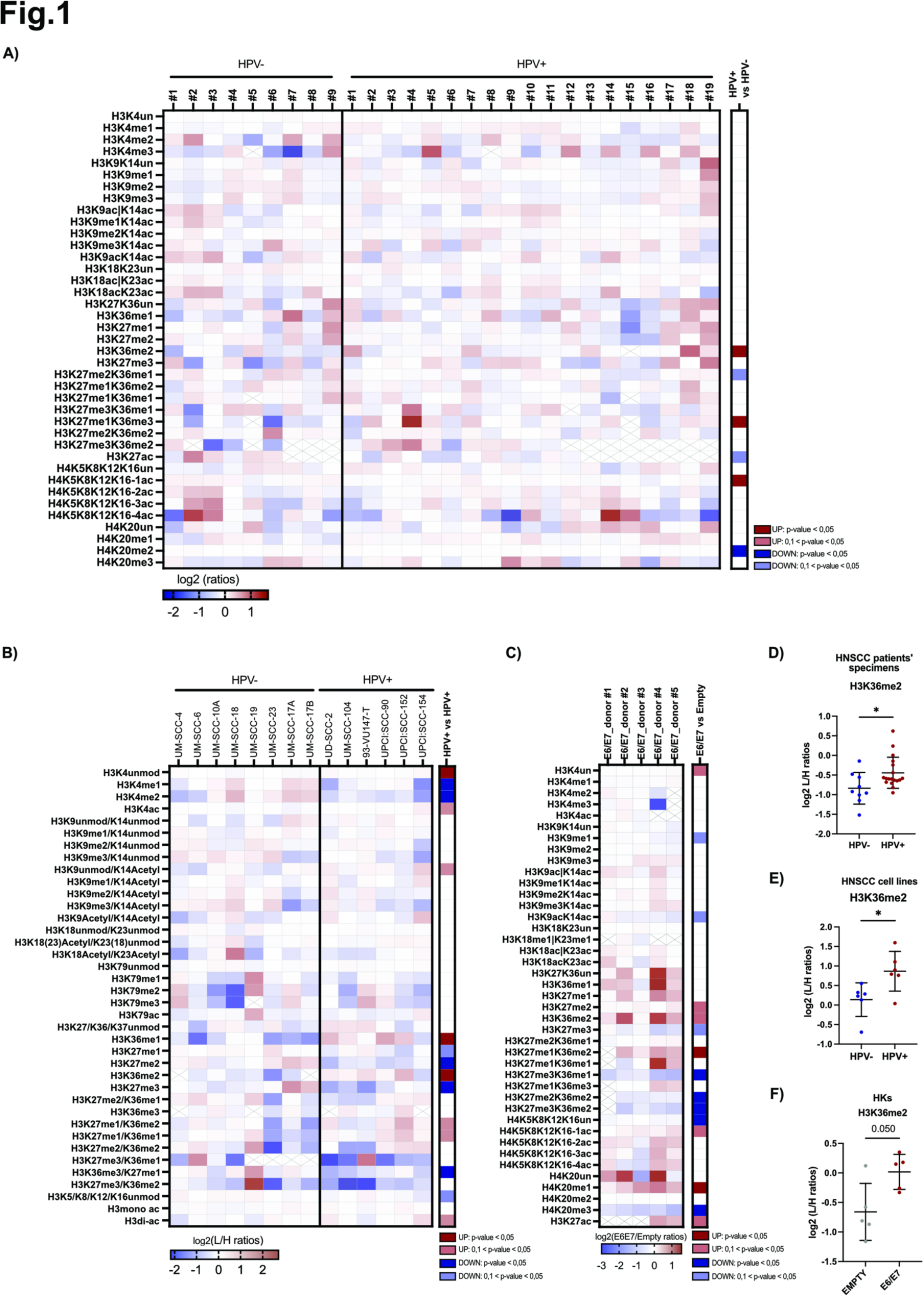
MS-based profiling of histone PTMs in HPV- and HPV + HNSCC samples. **A-B)** Heatmaps showing the levels of the indicated differentially modified histone peptides, which are expressed as L/H ratios (where L = sample and H = internal standard). The hPTMs were normalized to the average values across the samples. Crossed boxes represent non-assessed values. The panel on the right, with its related color legend, indicates the statistically significant differences in HPV + vs. HPV- samples, calculated through an unpaired Mann-Whitney test. hPTMs analyzed in 9 HPV- and 19 HPV + HNSCC FFPE patients’ tissue samples and 8 HPV- and 67 HPV + HNSCC cell lines are respectively shown in Figure A and B. **C)** hPTMs analysis was performed on Human Primary keratinocytes (HKs) derived from the healthy skin of 5 different donors and transduced with empty and HPV-16 E6/E7 encoding plasmid. Heatmap showing the L/H ratios for the indicated differentially modified histone peptides for E6/E7 overexpressing HKs normalized over the empty control. Crossed boxes represent non-assessed values. The panel on the right, with its related color legend, indicates significant or close to significant differences for each hPTM in E6/E7 overexpressing HKs vs. EMPTY control by paired t-test. **D-F)** Graphs showing the H3K36me2 levels detected by MS in HPV- HNSCC and HPV + HNSCC FFPE patients’ tissue samples; Unpaired Mann-Whitney test **(D)**, in HPV- HNSCC and HPV + HNSCC cell lines; Unpaired t-test **(E)**, and in HKs transduced with the empty or E6/E7 overexpressing plasmid; paired t-test **(F)**. Values are shown as log2 of L/H ratios. * p < 0,05.

To comprehensively characterize the hPTMs profiles in HPV+ and HPV- HNSCC, we employed the super-SILAC quantitative mass spectrometry (MS) approach. We analyzed both formalin-fixed paraffin-embedded (FFPE) HNSCC patient’s tissue samples and HNSCC cell lines (Fig. 1A-B). Since HPV-driven HNSCC mainly occur in the oropharynx [3, 4, 59], we focused on this anatomical subsite, and collected 19 HPV+ OPSCC, and 9 HPV- OPSCC FFPE samples (Table S4). All samples were processed according to previously established protocols [60, 61] and to ensure higher specificity, tumoral areas from FFPE slides were manually macrodissected. As shown in Fig. 1A, the analysis revealed considerable interpatient heterogeneity, with relatively few significantly different hPTMs between the HPV+ and HPV- subtypes. These included H3K36me2, H4K20me2, H3K27me1/K36me3 and the mono-acetylated form of histone H4. In particular, H3K36me2 —which is known to be upregulated in several cancer types and associated with oncogenic properties— was significantly enriched in HPV-positive compared to HPV-negative HNSCC samples. (Fig. 1A, D) [21, 29]. To evaluate whether HNSCC cell lines reflect the epigenetic landscape of the disease, we analyzed the hPTM profiles of 8 HPV-negative and 6 HPV-positive HNSCC cell lines using MS. Consistently with data obtained from patients’ specimens, our results revealed high heterogeneity across cell lines and some hPTMs being differentially enriched between the two subtypes (Fig. 1B). Strikingly, also in the cellular context, we confirmed that the main differences between the two subtypes occur at the H3K36 residues, with HPV+ HNSCC cell lines exhibiting significantly higher levels of H3K36me2 compared to the HPV-. Moreover, in HPV+ cell lines we observed increase of H3K36me1, and lower levels of H3K27me2 and H3K27me3 (Fig. 1B, E). Additional methylation differences in H3K36 and H3K27 exhibited trends approaching statistical significance (Fig. 1B).

To investigate whether the observed differences between HPV+ and HPV- HNSCC samples are driven by HPV16 E6/E7 oncoproteins, we stably infected HKs derived from five healthy donors, with retroviral vectors expressing HPV16 E6/E7. E6/E7 transduction was validated by western blot analysis showing the levels of p53 (Fig. S1A). Indeed, p53 stabilization was used as a surrogate marker of E6, due to the lack of E6 well-working antibodies.. The hPTMs profiles were subsequently characterized by MS (Fig. 1C). Our data revealed that E6/E7 profoundly affect the epigenomic landscape of the host cell. Interestingly, again the major differences again occur in the methylation levels of H3K36/H3K27 histone residues, with H3K36me2 being upregulated by E6/E7 expression (Fig. 1C, F), a finding further corroborated by WB analysis (Fig. S1B). In addition to that, E6/E7 also decreased H3K27me3 levels (although only near to significance), as observed in HNSCC cell lines, and induced the downregulation of H3K20me3 and the upregulation of H4K20me1 (Fig. 1C). Overall, our findings highlight previously unknown differences in H3K36 methylation levels between HPV+ and HPV- HNSCC, both in patient samples and cell lines. This hPTM could represent a novel clinical biomarker to discriminate the two HNSCC subtypes.

Moreover, our data offer the first comprehensive characterization of E6/E7-induced hPTMs alterations in host cells, providing the first evidence of their role in the upregulation of H3K36me2 levels. These findings support the involvement of E6/E7 in the epigenetic dysregulation observed in HPV+ HNSCC, suggesting that the elevated H3K36me2 levels may contribute to the pathogenesis of these tumors.

### HPV16 E6/E7 upregulate NSD2, a H3K36-specific histone methyltransferase, in primary keratinocytes and HNSCC cell lines

**Fig. 2:**
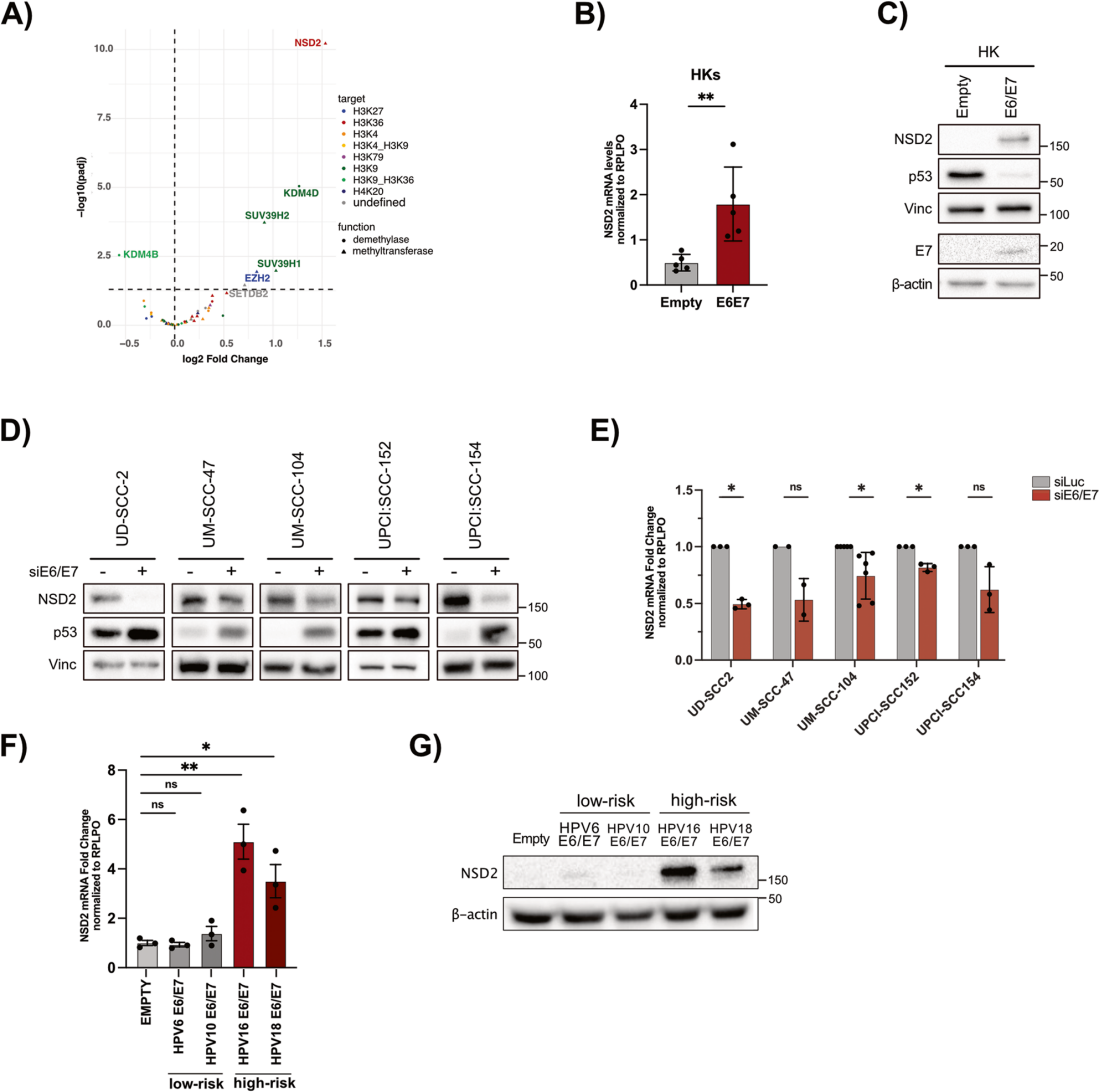
HR-HPV E6/E7 regulate NSD2 expression levels in HKs and HNSCC cell lines.** A-C)** HKs were transduced with empty or HPV-16 E6/E7 encoding vectors and harvested after selection. **A)** Total RNA was extracted and RNA-seq was performed on HKs derived from 4 donors and transduced with HPV16-E6/E7. The Volcano Plot shows the log2FC values (over the empty control) of the major known histone modifiers. The legends on the right indicate the main known targets (colors) and the function (shape) of the plotted histone modifiers. For the most strongly regulated genes, the corresponding names are reported in the figure. **B)** Total RNA was extracted and analyzed by RT-qPCR. The histogram shows the mRNA levels of NSD2 normalized on the housekeeping gene RPLP0. Values of 5 independent experiments, normalized relative to the empty group average, are expressed as means ± SD. Unpaired t-test with Welch’s correction. **C)** Western blot showing NSD2 levels in E6/E7-transduced HKs. TP53 was used as a surrogate marker of E6 overexpression. Vinculin and β-actin were used as loading controls. **D-E)** HPV + HNSCC cell lines were transfected with siE6/E7 or siLuc control and harvested after 72 h. **D)** Lysates were processed and analyzed by immunoblot with the indicated antibodies. Vinculin was used as a loading control. **E** Total RNA was extracted and analysed by RT-qPCR. For each cell line NSD2 mRNA levels were normalized on the RPLP0 housekeeping gene and expressed as means (± SD) of fold changes of at least 2 independent experiments. One sample t-test. **F-G)** HKs were transduced with the recombinant retroviral vector pLXSN encoding for the E6/E7 of low risk (HPV-6, HPV-10) and high-risk (HPV-16, HPV-18) HPV genotypes or with the empty vector as a control. **F)** Histogram showing the mRNA levels of NSD2 normalized on the housekeeping gene RPLP0. Values of 3 independent experiments, normalized relative to the empty control group average, are expressed as means ± SD. Unpaired t-test. **G)** Lysates were processed and NSD2 protein levels were analyzed by immunoblot. β-actin was used as a loading control. * *p* < 0,05; ** *p* < 0,01; ns, not significant

Alterations in hPTMs are frequently driven by mutations or dysregulation of epigenetic modifiers [17]. It is also well-known that HPV-E6/E7 interact with several epigenetic regulators and affecting their expression and activity [14–16]. Thus, we sought to explore whether E6/E7 modulates H3K36me2 deposition by altering the expression of specific histone-modifying enzymes. To this purpose, we performed RNA sequencing on primary HKs derived from four healthy donors and transduced with HPV16 E6/E7. Gene Set Enrichment Analysis (GSEA) performed on differentially enriched genes validated the system (Fig. S1C). Interestingly, differential gene expression analysis identified NSD2, a methyltransferase involved in H3K36me2 regulation, as one of the most significantly upregulated histone modifiers (Fig. 2A). The upregulation of NSD2 induced by E6/E7 was further validated by RT-qPCR and Western blotting (Fig. 2B, C). E6 and E7 overexpression were validated by Western Blot analysis (Fig. 2C).

To confirm E6/E7 role in NSD2 regulation, we silenced E6/E7 in HPV+ HNSCC cell lines using RNA interference (RNAi). E6/E7 knockdown was validated by RT-qPCR (Fig. S1D) and Western blotting (Fig. 2D). Silencing E6/E7 resulted in a significant downregulation of NSD2 at both protein and mRNA levels (Fig. 2D, E), further confirming that NSD2 is transcriptionally regulated by HPV16 E6/E7.

It is widely known that E6 and E7 proteins from different HPV genotypes exhibit distinct amino acid sequences and biological effects [62]. To determine whether NSD2 upregulation is mediated only by high-risk HPVs, we transduced HKs with E6/E7 constructs from low-risk (HPV6 and HPV10) and high-risk (HPV16 and HPV18). RT-qPCR confirmed their expression (Fig. S1E). Interestingly, only high-risk HPV16/18 E6/E7, and not the low-risk HPV types, induced NSD2 upregulation at both the mRNA and protein levels (Fig. 2F, G), raising the possibility that NSD2 may be involved in HPV-driven oncogenesis.

This represents the first evidence linking NSD2 regulation to HPV16/18 E6/E7 oncoviral proteins.

### NSD2 is overexpressed in HPV+ HNSCC cell lines and patient tissue samples

**Fig. 3:**
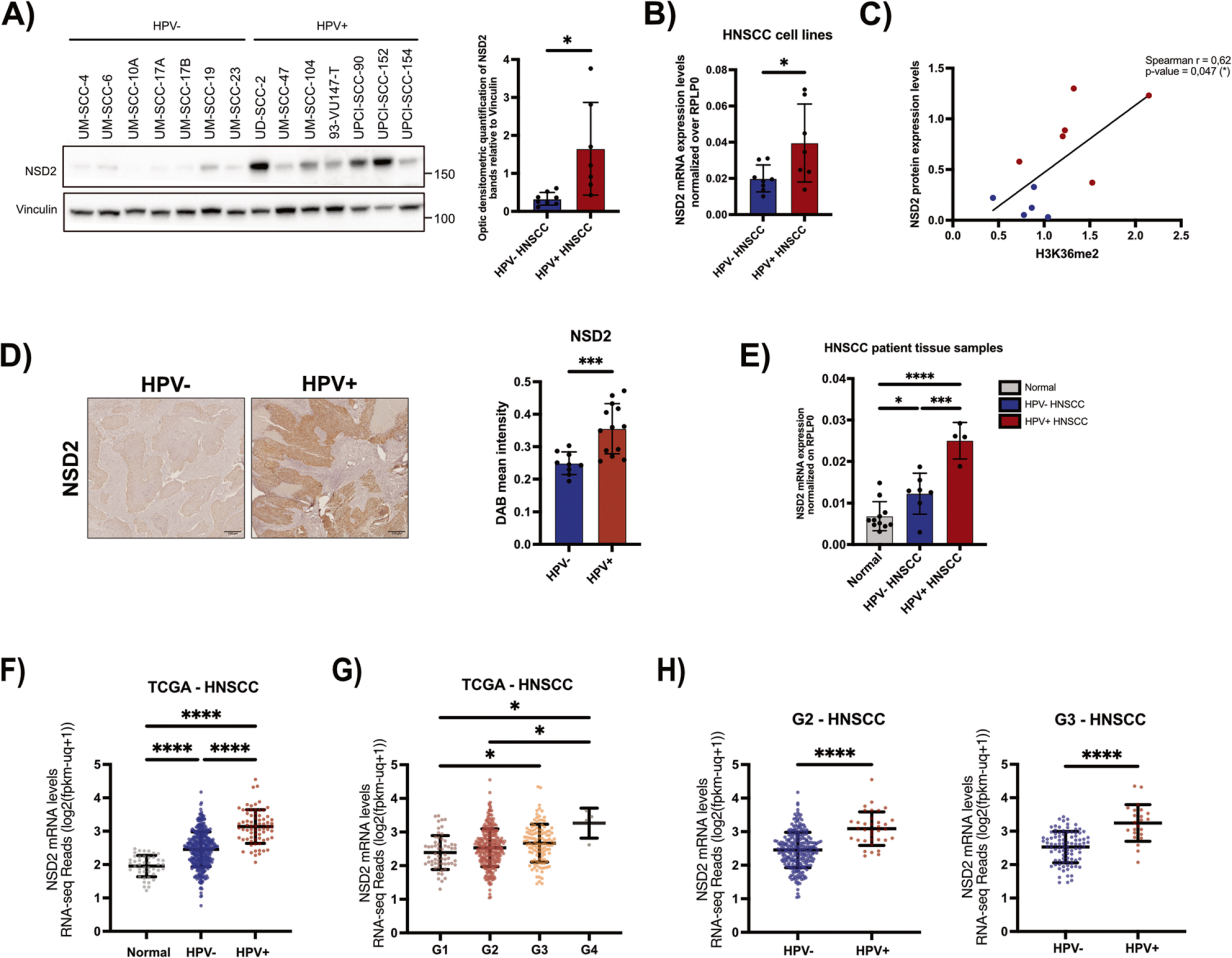
NSD2 expression levels in HNSCC cell lines and patients’ tissue samples.** A)** Immunoblot showing the NSD2 protein levels in 7 HPV- and 7 HPV+ HNSCC cell lines. Vinculin was used as a loading control. The histogram on the right shows the optical densitometric quantification of NSD2 bands normalized to Vinculin. Plotted results are the average of three independent experiments (± SD). Unpaired t-test with Welch’s correction. **B)** Total mRNA was extracted from 8 HPV- (the same of Fig. 3A plus UM-SCC-18) and 7 HPV+ HNSCC cell lines.The histogram shows the NSD2 mRNA levels of each group, analyzed by RT-qPCR and normalized on the housekeeping gene RPLP0. Each dot represent the average value of three independent replicates of each cell line. Values are expressed as means ± SD. Unpaired t-test. **C)** Correlation between the H3K36me2 levels, measured by MS, and NSD2 levels, measured by densitometric quantification of immunoblot bands, of HPV- and HPV+ HNSCC cell lines: Spearman correlation coefficient *r* = 0,62; p-value = 0,047. **D)** Representative images of NSD2 immunohistochemistry performed on 9 HPV- and 13 HPV+ HNSCC patients’ specimens. The histogram on the right shows the quantification of NSD2 signal, measured as DAB mean intensity. Values are expressed as means ± SD. Unpaired t-test. **E)** Total mRNA was extracted from 11 normal, 7 HPV- and 4 HPV+ HNSCC cryopreserved specimens. The histogram shows the NSD2 mRNA levels of each group, analyzed by RT-qPCR and normalized on the housekeeping gene RPLP0. Values are expressed as means ± SD. one-way ANOVA, multiple comparison test. **F)** NSD2 expression levels of 44 normal, 415 HPV- and 72 HPV+ HNSCC specimens from the TCGA Pancancer 2018 dataset. Data were downloaded from the “GDC Data Portal”. Values are expressed as means ± SD. one-way ANOVA multiple comparison test. **G)** NSD2 levels of HNSCC specimens from TCGA Pancancer 2018 dataset, clusterized according to the histological grade (G1, G2, G3, G4). Values are expressed as means ± SD. one-way ANOVA multiple comparison test. **H)** NSD2 levels of HPV- and HPV+ HNSCC specimens from TCGA Pancancer 2018 dataset, clusterized according to the histological grade (G2, on the left; G3, on the right). Values are expressed as means ± SD. Unpaired t-test. *, *p* ≤ 0,05; ** *p* < 0,01; *** *p* < 0,001; ****, *p* ≤ 0,0001; ns, not significant

Our results demonstrated that H3K36me2 is upregulated in HPV context, and that E6/E7 oncoprotein specifically upregulate NSD2, the main regulator of this histone mark. This prompted us to assess whether NSD2 expression levels differed in HPV+ and HPV- HNSCC. To measure NSD2 levels, we performed WB analysis on a panel of 7 HPV+ and 7 HPV- HNSCC cell lines (Fig. 3A). Our results indicate that NSD2 is significantly overexpressed in HPV+ HNSCC subtype compared to the HPV- one (Fig. 3A). These data were also confirmed at the mRNA levels, as shown in Fig. 3B. Densitometric analysis of immunoblots revealed a positive correlation between NSD2 levels and global H3K36me2 enrichment, quantified by MS (Spearman’s *r* = 0.62, *p* = 0.047) (Fig. 3C). To corroborate our analysis on patients’ samples, we performed immunohistochemistry (IHC) on 9 HPV- and 13 HPV + HNSCC FFPE tumor tissue samples (Fig. 3D). Notably, the majority of the samples analyzed by IHC overlapped with those previously analysed by Mass Spectrometry (Fig. 1A). Coherently with cell lines data, also in patients we observed a significant upregulation of NSD2 in the HPV+ subtype compared to HPV- one. Moreover, NSD2 mRNA expression levels were assessed in 12 cryopreserved HNSCC tissues (4 HPV+ and 7 HPV-), alongside their normal counterparts. To expand the analysis to a larger cohort, we interrogated the TCGA database, classifying 523 patients based on HPV status (415 HPV- HNSCC, 72 HPV+ HNSCC and 44 Normal) [57, 58]. Interestingly, all the analyses confirmed not only that NSD2 is overexpressed in HPV+ tumors compared to the HPV-, but also in both the HNSCC subtypes compared to normal tissues (Fig. 3D-F), as previously observed [20, 24]. In addition, TCGA data analysis revealed that NSD2 expression correlates with higher histological grade (Fig. 3G). Since HPV+ HNSCC are generally poorly differentiated and characterized by higher histopathological grading than the HPV- ones, we examined whether NSD2 overexpression in HPV+ tumors was merely a grade-dependent consequence. Stratification by histological grade (G2 and G3) revealed that NSD2 remained upregulated in HPV+ tumors regardless of differentiation status, reinforcing the role of HPV E6/E7 in NSD2 regulation (Fig. 3H).

Furthermore, TCGA data showed that genetic alterations in NSD2 are rare in HNSCC, with only 3% of patients harboring mutations (including missense, truncating, fusions, and deep deletions), suggesting that, in HNSCC, NSD2 dysregulation is primarily due to overexpression rather than genetic mutations [57, 58].

Overall, our data demonstrate that NSD2 is upregulated in HNSCC, with even higher expression in HPV + cases in both patients and cell lines. These findings support the previously described HPV E6/E7-mediated transcriptional regulation and suggest that HNSCC cell lines accurately model NSD2 expression patterns observed in patient tumors, making them valuable models for epigenetic research.

### NSD2 downregulation reduces cell proliferation and EMT in both HPV+ and HPV- HNSCC

Given the distinct clinicopathological and molecular features of HPV+ and HPV- subtypes, we hypothesized that targeting NSD2 could elicit different tumor-suppressive effects based on the HPV status.

**Fig. 4:**
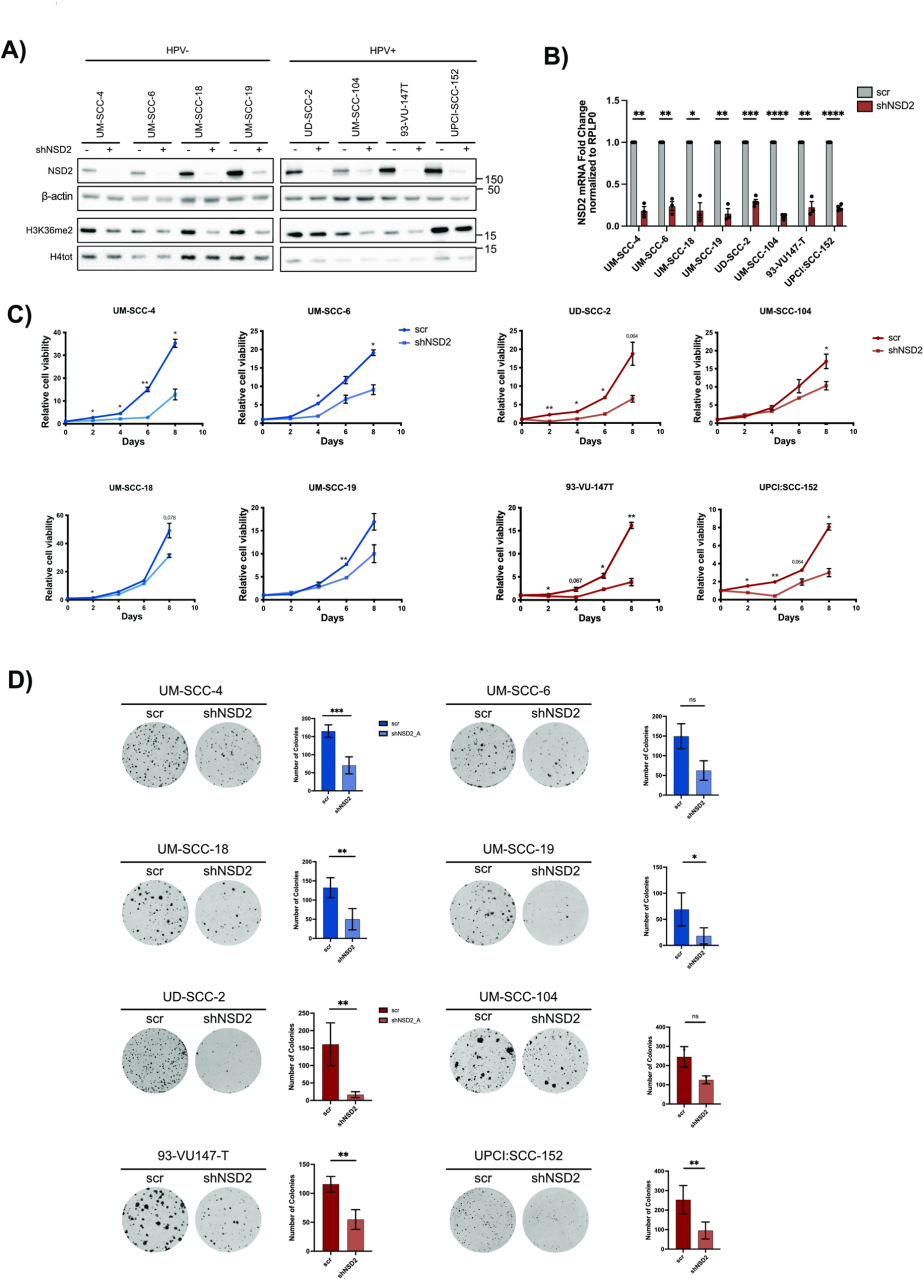
Silencing NSD2 reduces cell proliferation in HPV + and HPV- HNSCC cell lines. 4 HPV- and 4 HPV + HNSCC cell lines were transduced with shNSD2 (_A) and scrambled control. **A)** Immunoblots showing NSD2 and H3K36me2 protein levels in HNSCC cell lines upon NSD2 silencing. β-actin and H4 total were respectively used as housekeeping controls. **B)** Graph showing NSD2 mRNA levels of NSD2-silenced HNSCC cell lines, analyzed by RT-qPCR and normalized on the RPLP0 housekeeping gene. Values are represented as fold changes on the scrambled control and expressed as means ± SD. One-sample t-test. **C)** Cell proliferation was assessed on 4 HPV- (in blue) and 4 HPV+ (in red) NSD2-silenced HNSCC cell lines. Cells were counted at day 0, 2, 4, 6 and 8. Results of at least two replicates are shown. Unpaired t-test. **D)** Colony Formation Assay performed on 4 HPV- (in blue) and 4 HPV+ (in red) NSD2-silenced HNSCC cell lines. Representative images are reported for each condition and the respective histograms on the right show the quantified number of colonies/well. Results are the average of at least two replicates. Unpaired t-test. *, p ≤ 0,05; ** p < 0,01; *** p < 0,001; ****, p ≤ 0,0001; ns, not significant.

To assess the NSD2’s oncogenic role, we stably knocked down (KD) NSD2, using two shRNA constructs (shNSD2_A and shNSD2_B), in a panel of HPV+ and HPV- HNSCC cell lines. The shRNAs target two NSD2 isoforms (specifically called MMSETI and MMSETII) but not the shorter isoform REIIBP, which is anyway not implicated in H3K36me2 regulation [63]. Hereafter, shNSD2_A was used in the main figures and is referred to as shNSD2 for simplicity, whereas validations performed with shNSD2_B are presented in the supplementary figures. NSD2 knockdown effectively reduced both mRNA and protein levels of NSD2, leading to a significant reduction in H3K36me2 levels (Fig. 4A-B, S2A).

Next, we evaluated cell proliferation across 4 HPV- and 4 HPV+ HNSCC cell lines. NSD2 knockdown significantly reduced proliferation in both subtypes (Fig. 4C; S2B). Colony formation assays further supported these data, as shown in Fig. 4C and S2C.

In other tumor systems, it was shown that NSD2 is implicated in promoting EMT and cell migration [22, 29]. Thus, we asked whether NSD2 exerts similar function in HNSCC.

**Fig. 5:**
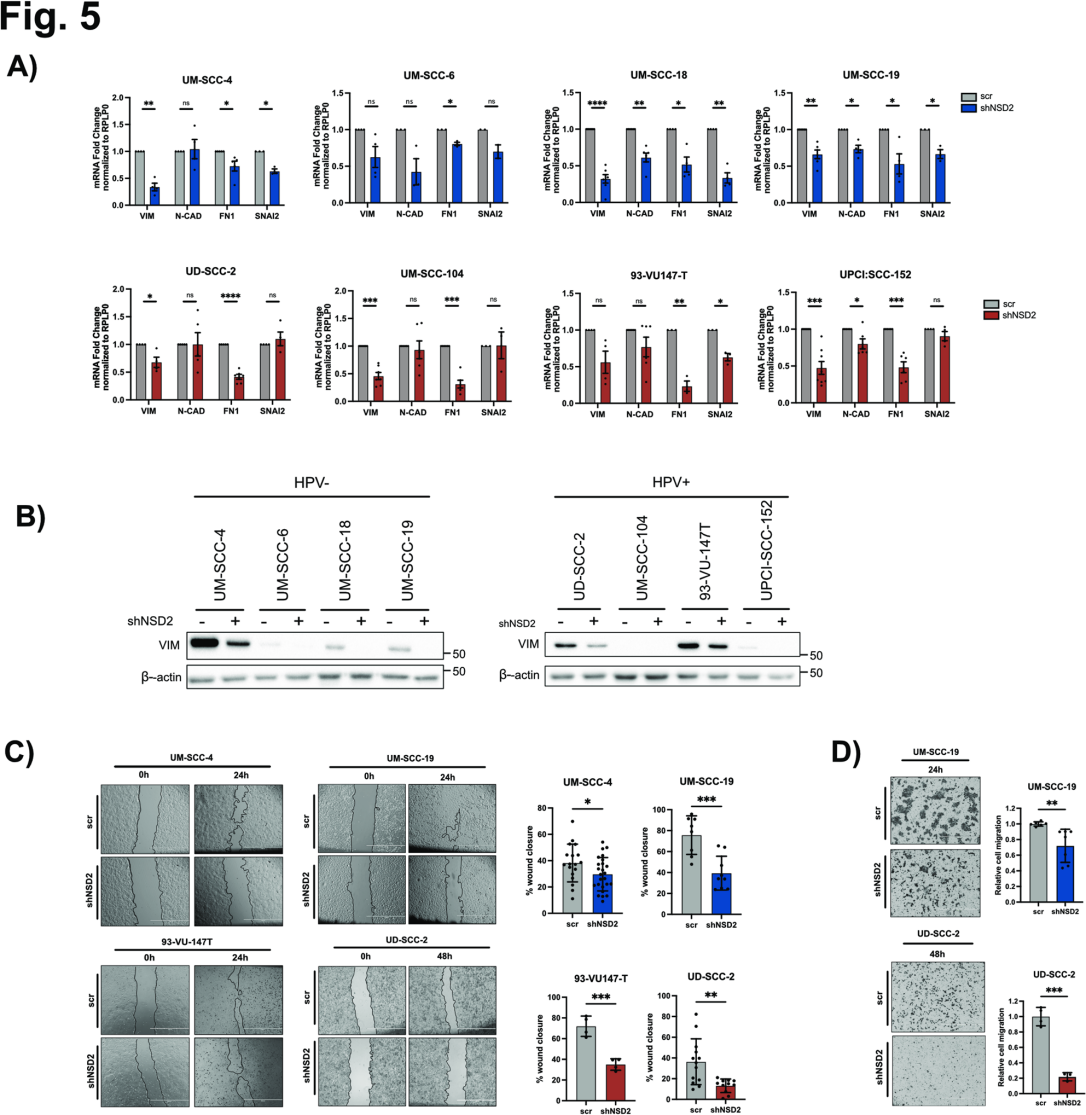
Silencing NSD2 reduces cell migration in HPV + and HPV- HNSCC cell lines. 4 HPV- and 4 HPV + HNSCC cell lines were transduced with shNSD2 (_A) and scrambled control.** A)**Histograms show the mRNA levels of mesenchymal markers (Vimentin, N-cadherin, Fibronectin and Snai2), analyzed by RT-qPCR, in 4 HPV- (in blue) and 4 HPV+ (in red) HNSCC cell lines. Data were normalized on RPLP0. Values of at least two independent experiments are represented as fold changes on the scrambled control and expressed as means ± SD. One-sample t-test. **B)** Immunoblots showing Vimentin levels in 4 HPV- and 4 HPV+ NSD2-silenced HNSCC cell lines. β-actin was used as housekeeping. **C)** Representative phase contrast pictures of wound healing assays at time 0 and 24 or 48 h, on two HPV- (in blue) and two HPV+ (in red) HNSCC upon NSD2 silencing. Graphs represent the percentage of wound closure expressed as means ± SD. For each cell line values were obtained from at least three replicates and normalized relative to the scrambled group average. Unpaired t-test. **D)** Representative phase contrast pictures of transwell migration assays performed on an HPV- and an HPV + HNSCC cell line upon NSD2-silencing. Cells were fixed and stained respectively after 24–48 h. Graphs represent the quantified migrated cells: for each cell line values were obtained from at least two replicates and normalized relative to the scrambled group average. Values are expressed as means ± SD. Unpaired t-test. *, p ≤ 0,05; **, p ≤ 0,01; ***, p ≤ 0,001; ****, p ≤ 0,0001; ns, not significant (t-test).

To evaluate EMT promotion, we silenced NSD2 in 4 HPV- and 4 HPV+ HNSCC cell lines. Independently on the HPV status, NSD2 KD reduced the expression of some EMT markers (Vimentin, N-cadherin, Fibronectin, SNAI2) either at mRNA or protein levels (Fig. 5A-B; S2D).

Moreover, to evaluate the role of NSD2 in cell migration, we silenced NSD2 in 2 HPV- and 2 HPV + cell lines and performed wound healing and transwell migration assays. Coherently with EMT data, we demonstrated that NSD2-KD decreased migratory potential in both HPV- and HPV+ cell lines (Fig. 5C-D; S2E).

**Fig. 6:**
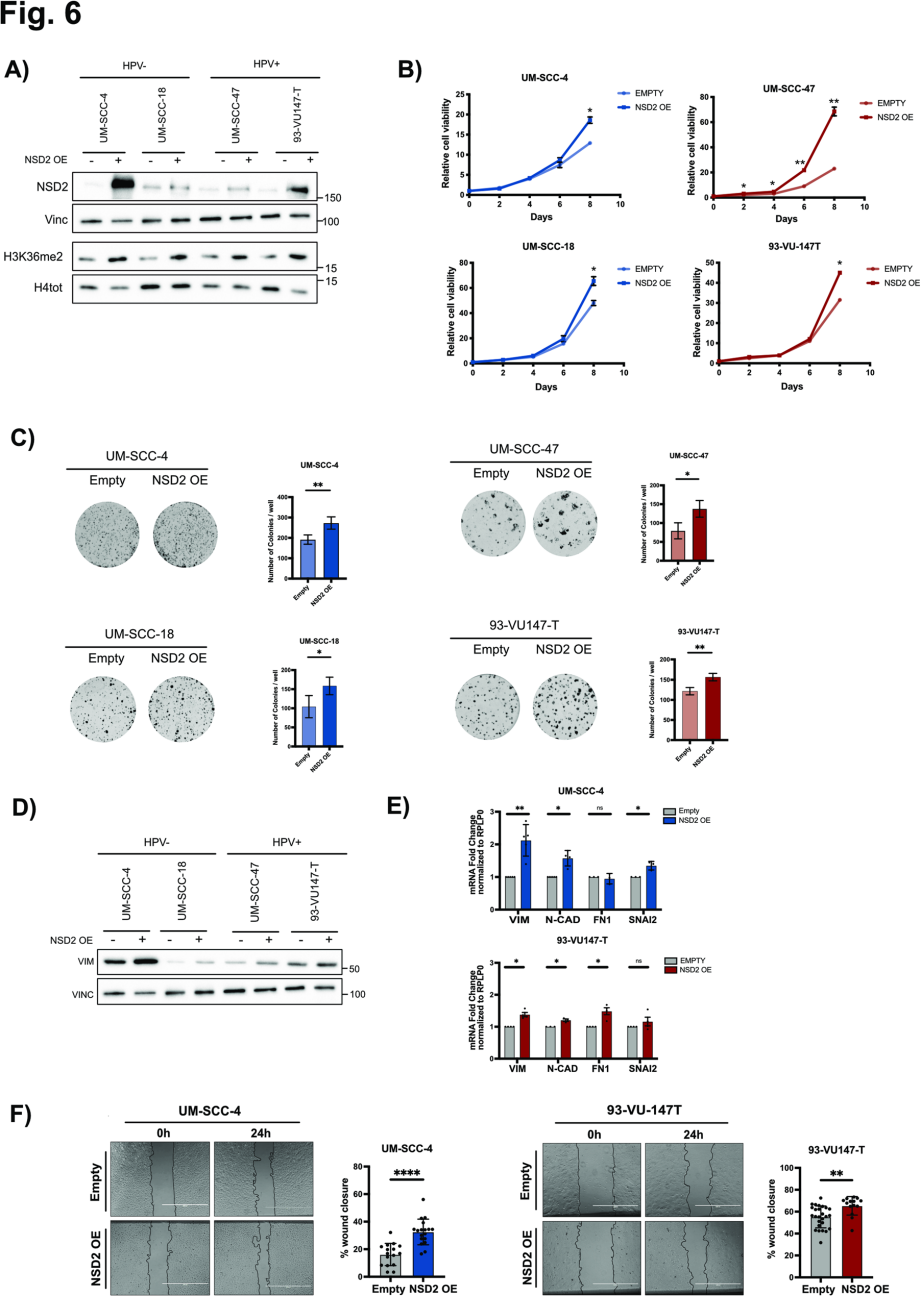
FNSD2 overexpression increases cell proliferation and cell migration in HPV + and HPV- HNSCC cell lines. NSD2 was overexpressed in 2 HPV- (UM-SCC-4, UM-SCC-18) and 2 HPV+ (UM-SCC-47, 93-VU147-T). **A) **Western Blots showing the NSD2 and H3K36me2 protein levels in 2 HPV- and 2 HPV + HNSCC cell lines upon NSD2 overexpression. Vinculin and H4 total were used as loading controls. **B)** Cell proliferation has been assessed in 2 HPV- (in blue) and 2 HPV+ (in red) HNSCC cell lines upon NSD2 overexpression. Cells were counted every 2 days for five time points (day 0, 2, 4, 6, 8). Values of two replicates are shown. Unpaired t-test. **C) **Colony Formation Assay performed on 2 HPV- (in blue) and 2 HPV+ (in red) NSD2-overexpressing HNSCC cell lines. Representative images are reported for each condition and the respective histograms on the right, show the quantified number of colonies/well. Results are the average of at least two replicates. Unpaired t-test. **D)** Western Blots showing the Vimentin protein levels in 2 HPV- and 2 HPV + HNSCC cell lines, upon NSD2 overexpression. Vinculin was use as a loading control. **E)** Total mRNA was extracted from an HPV- (UM-SCC-4) -in blue- and an HPV+ (93-VU147-T) -in red- HNSCC cell line upon NSD2 overexpression. Histogram show the mRNA expression levels of Vimentin, N-cadherin, Fibronectin and Snai2 analyzed by RT-qPCR and normalized on the RPLP0 housekeeping gene. Values of at least three independent experiments are represented as fold changes on the empty vector control and expressed as means ± SD. One-sample t-test. **F)** Phase contrast representative pictures of wound healing assays performed on an HPV- (UM-SCC-4) and an HPV+ (93-VU147T) HNSCC cell line transduced with the empty or the NSD2-overexpressing vector. Images were acquired at time 0 and 24 h. Graphs on the right represent the percentage of wound closure. For each cell line values from at least three replicates were normalized relative to the empty group average and are expressed as means ± SD. Unpaired t-test. *, p ≤ 0,05; **, p ≤ 0,01; ***, p ≤ 0,001; ****, p ≤ 0,0001 (t-test).

To corroborate our results, we also assessed NSD2 overexpression (OE) in 2 HPV- and 2 HPV + HNSCC cell lines. The overexpression of NSD2 and the consequent increase of H3K36me2 levels were validated by WB (Fig. 6A). Coherently with NSD2 KD results, we observed that NSD2-OE prompted higher proliferation rates and increased colony formation potential (Fig. 6B-C). Moreover, it modulated the expression of EMT markers leading to the upregulation of mesenchymal markers (Fig. 6D, E) and increased cell migration in both an HPV- and an HPV+ cell line as assessed by transwell migration assay (Fig. 6F).

Overall, these results indicate that NSD2 promotes cell proliferation, EMT, and cell migration in HNSCC, irrespective of HPV status.

### Transcriptomic analysis reveals that NSD2 silencing affects common and distinct pathways in HPV+ and HPV- HNSCC cell lines, promoting cell differentiation in the HPV+ subtype

**Fig. 7:**
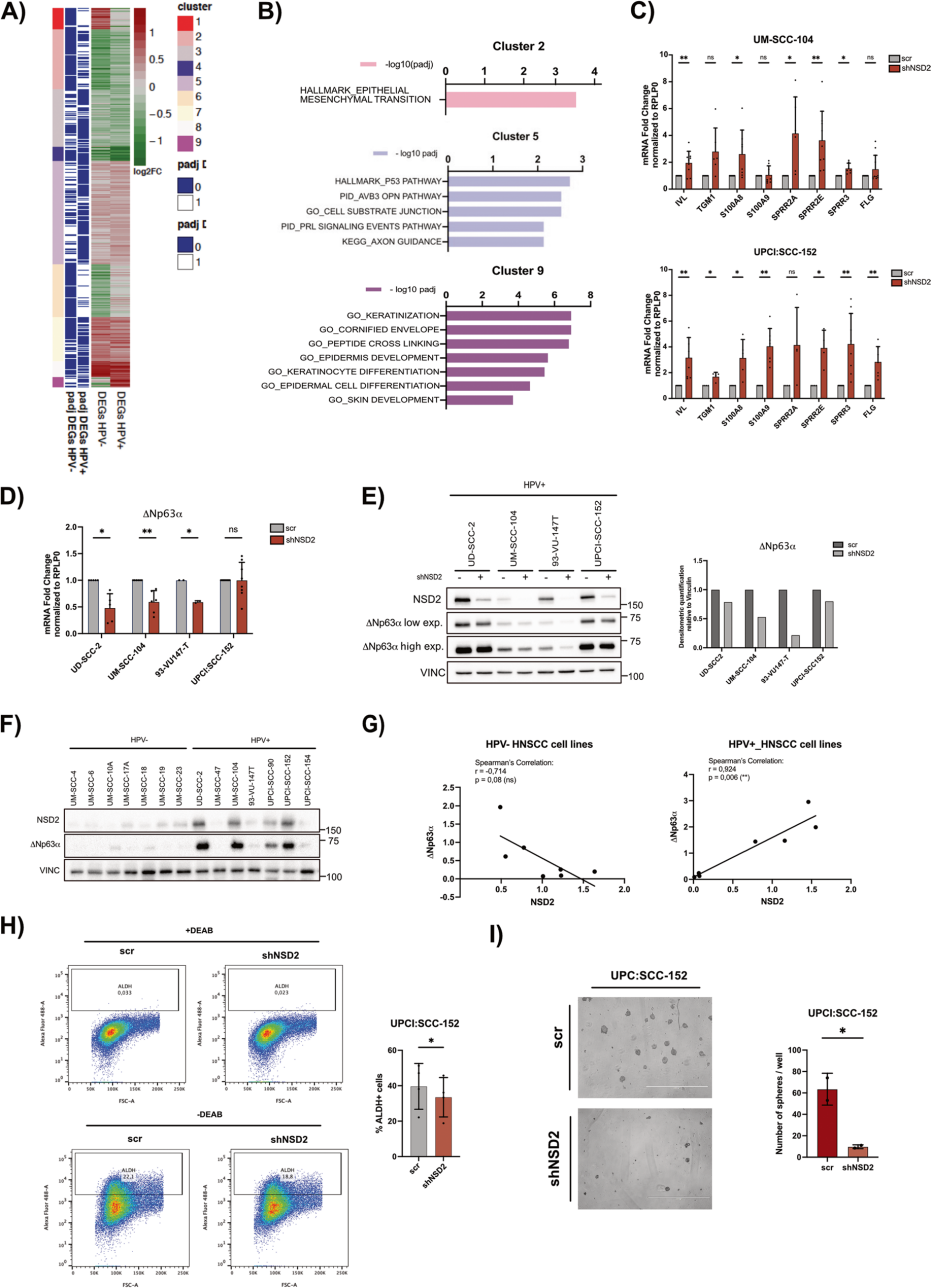
NSD2 silencing affects common and distinct pathways in HPV+ and HPV- HNSCC cell lines.** A)** RNA-seq was performed on 4 HPV- (UM-SCC-4, UM-SCC-6, UM-SCC-18, UM-SCC-19) and 3 HPV+ (UD-SCC-2, UM-SCC-104, UPCI: SCC-152) HNSCC cell lines transduced with shNSD2_A or scr control. Heatmap showing the DEGs in HPV- and HP+ HSNCC cell lines upon NSD2 silencing. K-means clustering has been applied with a number of clusters k = 9. The red-green scale represents the log2FC values, while the blue and white boxes indicate if the gene is statistically significant or not, respectively, for each of the two subgroups. Color legend indicates the number of each cluster. **B)** Gene Ontology (GO) analysis was performed on identified clusters. Bar plots show significant gene ontologies enriched in Cluster 2, 5 and 9. **C)** Total mRNA was extracted from the HPV+ HNSCC cell lines, UM-SCC-104 and UPCI:SCC-152, upon NSD2 silencing. Histograms showing the mRNA expression levels of a panel of differentiation markers analyzed by RT-qPCR and normalized on RPLP0. Values of at least three independent experiments are represented as fold changes on the scrambled control and expressed as means ± SD. One-sample t-test. **D)** Graph showing the ΔNp63α mRNA expression levels, analyzed by RT-qPCR and normalized on RPLP0. Values of at least two independent experiments, represented as fold changes on the scrambled control, are expressed as means ± SD. One-sample t-test. **E)** Immunoblot showing ΔNp63α protein levels in 4 HPV + HNSCC cell lines, upon NSD2 silencing. To optimize the visualization, both low exposure and high exposure acquisition are shown. Vinculin was used as loading control. The histogram on the right, shows the optical densitometric quantification of ΔNp63α bands normalized on Vinculin. **F)** Immunoblot showing NSD2 and ΔNp63α protein levels in 7 HPV- and 7 HPV+ HNSCC cell lines. Vinculin was used as loading control. **G)** Correlation between ΔNp63α and NSD2 protein levels measured by densitometric quantification of Western blot bands in HPV- (Spearman correlation coefficient *r*=−0,714; p-value = 0,08) and HPV+ HNSCC cell lines (Spearman correlation coefficient *r* = 0,924; p-value = 0,006). **H)** ALDH + cells were detected and quantified through the ALDEFLUOR assay in UPCI:SCC-152 cell line, upon NSD2-silencing, and acquired by FACS analysis. Treatment with DEAB inhibitor was used as negative control. The histogram shows the mean of four independent experiments ± SD. Paired t-test. **I)** Sphere formation assay was performed in UPC:SCC-152 cell line upon NSD2-silencing. Images were acquired 15 days post-plating; spheres > 70 μm in diameter were counted. The histogram shows the mean of two independent experiments ± SD. Unpaired t-test. *,*p* ≤ 0,05; **,*p* ≤ 0,01; ns, not significant (t-test).

To further investigate the NSD2 oncogenic role in HPV+ and HPV- HNSCC, we conducted RNA-seq analysis on 4 HPV- and 3 HPV+ HNSCC cell lines transduced with shNSD2 or negative scrambled control. Unsupervised cluster analysis of differentially expressed genes (DEGs) resulting from NSD2 knock-down in both HPV+ and HPV- groups, revealed nine distinct gene clusters. While most of them were characterized by similar regulatory patterns across the subtypes, Cluster 1 and 9, included genes uniquely affected in either HPV+ or HPV- cell lines (Fig. 7A). GO analysis performed on each cluster confirmed the NSD2’s involvement in EMT (Cluster 2) and cell-substrate junction regulation (Cluster 5) in both subtypes (Fig. 7B). Interestingly, genes of Cluster 9 were specifically upregulated in HPV+ cell lines and were enriched for GO terms related to epithelial differentiation, such as “Keratinocyte differentiation”, “Keratinization,” and “Epidermal cell differentiation” (Fig. 7A, B), suggesting a role for NSD2 in HPV-driven differentiation. Since computing the overall log2FC could potentially mask cell line-specific transcriptional regulation, we also reported in Fig.S3A an heatmap showing the log2FC of the same genes of Fig. 7A for each single cell line. This support the overall consistency among the cell lines of each subgroup following the NSD2 KD. Moreover, we then focused our attention on cluster 9 and compared the log2FC for each of the HPV+ cell lines with that of all the HPV- ones, and added a boxplot in Figure S3B. Despite appearing more variable compared to other clusters, the analysis still reveals a stronger upregulation of epithelial differentiation and keratinization genes in the HPV+ subtype.

Notably, differential gene expression analysis revealed 215 DEGs in HPV+ HNSCC cell lines and 415 DEGs in the HPV- (FDR < 0.05, |Log2FC| > 0.5) (Fig. S3C). The relatively modest number of DEGs identified in each subgroup could be attributed to the fact that only major transcriptional changes induced by NSD2-KD are shared across different cell lines. Also, only 9.7% of downregulated and 13.7% of upregulated genes overlapped between the two subtypes (Fig. S3C), suggesting not only a cell-type-dependent role for NSD2, but also a different effect according to the HPV status.

GSEA separately performed on DEGs from HPV- or HPV+ HNSCC cell lines, confirmed data from cluster analysis, showing that NSD2 regulates EMT and metastasis in both subtypes, while a more pronounced impact on epithelial differentiation in HPV+ cell lines (Fig. S3D-E). This is particularly noteworthy considering the higher histological grade and reduced keratinization generally characterizing HPV+ HNSCC compared to the HPV-, the known differentiation blockade induced by hr-HPVs and the described positive correlation between NSD2 and histological grade (Fig. 3G) [3, 4, 10, 11, 24]. Additionally, NSD2 has been implicated in regulating cellular differentiation and lineage plasticity in other biological contexts such as erythroid, lymphocyte and neural progenitor cell differentiation, osteogenesis and gametogenesis [30, 32, 64, 65]. Therefore, the NSD2-KD mediated induction of epithelial differentiation in HPV+ HNSCC, aligns with its broader role in modulating cell fate.

To validate the role of NSD2 in epithelial differentiation, we performed RT-qPCR analysis in two representative HPV+ HNSCC cell lines (UM-SCC-104 and UPCI:SCC-152). As expected, RT-qPCR analysis confirmed the upregulation of the mRNA levels of several markers of terminal differentiation (IVL, TGM1, S100A8, S100A9, SPRR2A, SPRR2E, SPRR3, FLG) upon NSD2 silencing (Fig. 7C). In accordance with RNA-seq data, the same analyses performed on three HPV- HNSCC cell lines showed a milder and less consistent increase of the same differentiation markers across the HPV- subtype cell lines (Fig.S4A). Together, our findings reinforce the oncogenic role of NSD2 in HNSCC, highlighting its involvement in EMT, and reveal a novel role in regulating epithelial cell differentiation, suggesting that targeting NSD2 could promote differentiation, particularly in HPV+ HNSCC.

### NSD2 regulates the oncogene and master regulator of epithelial differentiation, ΔNp63α

ΔNp63α, a key oncogene in HNSCC, is the predominant isoform of the p63 transcription factor expressed in epithelial stem cells and undifferentiated basal cells of the epithelium. It plays a crucial role in the development and maintenance of stratified epithelial tissues by promoting keratinocyte self-renewal and regulating key factors involved in growth and differentiation [66, 67]. Previous data from our lab have demonstrated that hr-HPV oncoproteins E6/E7 induce ΔNp63α overexpression, which is consequently upregulated in HPV+ tumors compared to the HPV- [68]. Since our data have clearly shown that E6/E7 mediates NSD2 upregulation, we asked whether NSD2 may also have an effect on ΔNp63α levels. Interestingly, we observed in a panel of 4 HPV+ HNSCC cell lines, that NSD2 silencing regulates ΔNp63α, reducing its levels both at the mRNA and protein levels (Fig. 7D-E). To investigate the correlation between NSD2 and ΔNp63α in HNSCC cell lines, we performed WB analysis on NSD2 and ΔNp63α in a panel of 7 HPV- and 7 HPV+ HNSCC cell lines. Interestingly, we observed a strong and significant correlation between NSD2 and ΔNp63α protein expression levels restricted only in HPV+ cell lines (Spearman *r* = 0,9424, *p* = 0,006) (Fig. 7F, G).

Moreover, given the role of ΔNp63α in maintaining the self-renewal capacity of epithelial stem cells [69], and considering that NSD2 regulates the expression of this master regulator, we wondered whether NSD2 silencing might also affect cell plasticity and stemness. To assess this phenotype, we took advantage of ALDH enzyme activity, assessed by ALDEFLUOR kit. In details, ALDH activity is a key feature of stem cells and in particular of cancer stem cells (CSCs) and in patients is highly correlated with higher tumor grade and worse prognosis [4, 70]. To verify that NSD2 silencing promotes an impairment on stemness proprieties, we silenced NSD2 in two HPV+ and two HPV- HNSCC cell line. Interestingly, the silencing promoted a decrease of ALDH activity in both the HPV+ cell lines (Fig. 7H, S4B), and consequently a decrease in the percentage of cancer stem cells, suggesting a possible reduction of the stem-like properties and self-renewal capacity of the cells. Conversely, in the tested HPV- HNSCC cell lines we observed more heterogeneous and less consistent results (Fig. S4B). These findings further suggest that the NSD2-mediated reduction of the stem-like population is more pronounced and consistent in the HPV + subtype. Notably, these data are well recapitulated and validated by results obtained performing the sphere formation assays on the same cell lines, as shown in Fig. 7I and S4C.

Taken together these findings suggest that NSD2 functions upstream of ΔNp63α and that the E6/E7-driven upregulation of ΔNp63α may be mediated by NSD2 overexpression. Given the crucial oncogenic role of ΔNp63α in HNSCC, these results hint that targeting NSD2 could provide an indirect strategy to modulate ΔNp63α levels, offering a novel therapeutic avenue.

### NSD2 regulates epithelial differentiation in HKs and HPV+ HNSCC

**Fig. 8:**
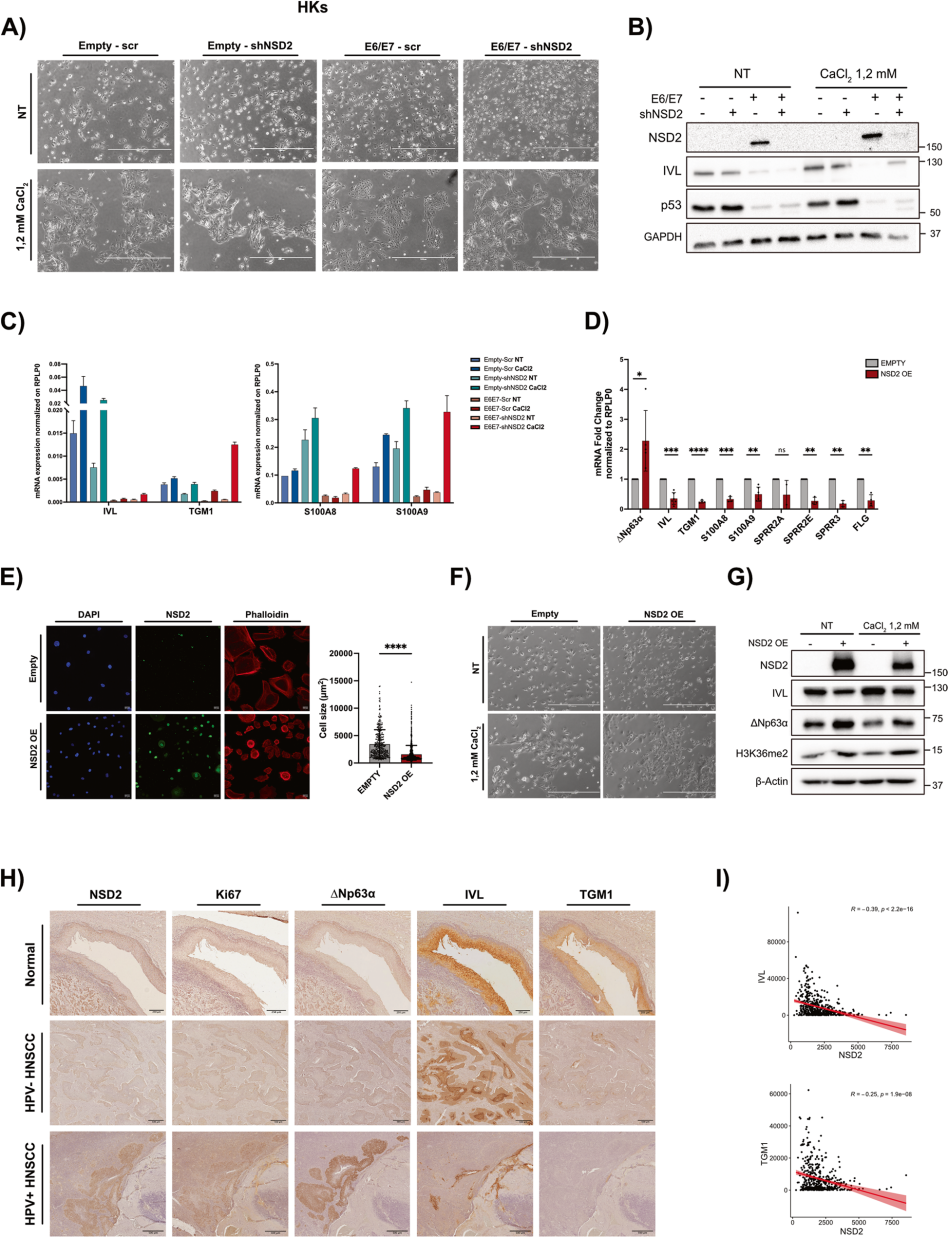
NSD2 regulates epithelial cell differentiation and mediates the E6/E7-induced differentiation blockade. **A-C)** HPV16-E6/E7 oncoviral proteins were overexpressed in Human Primary Keratinocytes, that were also transduced with shNSD2_A or scrambled control and treated with 1,2 mM CaCl_2_. **A) **Representative phase contrast images showing the morphological changes of HKs upon the indicated treatments. **B)** Immunoblot showing changes in NSD2 and IVL protein expression levels. P53 was used as a surrogate marker of E6 overexpression. GAPDH was used as loading control. **C)** Histograms showing the mRNA expression levels of the differentiation markers IVL, TGM1, S100A8 and S100A9, analyzed by RT-qPCR and normalized on RPLP0. Values of two technical replicates are expressed as means ± SD. **D)** Total mRNA was extracted from HKs overexpressing NSD2. The histogram shows the mRNA levels of ΔNp63α, IVL, TGM1, S100A8, S100A9, SPRR2A, SPRR2E, SPRR3 and FLG, analyzed by RT-qPCR and normalized on RPLP0. Values of at least three independent experiments are represented as fold changes on the empty vector control and expressed as means ± SD. One sample t-test. **E)** Representative confocal images of NSD2-overexpressing HKs. Phalloidin (red) was stained to show cell size and morphology, Nuclei were stained with DAPI (blue) and NSD2 with Alexa 488 (green). The graph shows the difference in cell size between NSD2-overexpressing HKs and control. Cells’ area was quantified using phalloidin and nuclei staining was used for cellular segmentation. Values are expressed as mean ± SD. Unpaired t-test. **F-G)** HKs transduced with the NSD2-encoding vector or with the empty control were treated with 1,2 mM CaCl_2_. **F)** Representative phase contrast pictures showing the morphological changes of NSD2-overexpressing HKs upon 1,2 mM CaCl_2_ treatment. **G)** Immunoblot showing changes in IVL, ΔNp63α, H3K36me2 protein levels. β-actin was used as loading control. **H)** Panel showing representative images of IHC performed on normal and HNSCC patients’ FFPE tissue samples and acquired with the Nanozoomer brightfield microscopy. Specimens were stained for NSD2, Ki67, ΔNp63α, IVL and TGM1. Both HPV- and HPV+ HNSCC cases are tumors of Grade3 (G3). **I)** RNA-seq data from TCGA Dataset were downloaded and analysed through the SRPlot software. Graphs showing the correlation between NSD2 and IVL or TGM1. Calculated Spearman correlation is −0,39 (*p* < 0,0001) for NSD2 and IVL and − 0,25 (*p* < 0,0001) for NSD2 and TGM1. *, p ≤ 0,05; **, p ≤ 0,01; ***, p ≤ 0,001; ****, p ≤ 0,0001 (t-test)

E6/E7 are well known to inhibit terminal epithelial differentiation [10, 11]. Indeed, we have demonstrated that these oncoviral proteins regulate NSD2 expression, which in turn, is implicated in epithelial differentiation. Thus, we investigated a possible link between E6/E7-driven NSD2 upregulation and the HPV-induced-impairment of keratinocyte differentiation. To investigate this aspect, we employed human primary keratinocytes (HKs) transduced with hr-HPV E6/E7 oncoproteins, and induced differentiation via increasing extracellular calcium (CaCl_2_). This treatment triggers notable morphological changes, such as increased cell-to-cell contacts and a more stretched and enlarged cell morphology, as shown in Fig. 8A and S5A [11, 71, 72]. Conversely, upon E6/E7 overexpression, CaCl_2_-mediated differentiation process is impaired, with more undifferentiated phenotype such as round-shaped cells and less cell-cell contacts (Fig. 8A, Fig.S5A). Finally, to test the role of NSD2 into the epithelial differentiation, we KD NSD2 in presence of E6/E7 overexpression. Strikingly, we observed that NSD2 KD restored CaCl_2_-induced differentiation, both morphologically and molecularly, effectively rescuing the phenotype impaired by E6/E7 expression (Fig. 8A-C, S5A, S6A-B). WB and RT-qPCR analyses revealed that NSD2-KD rescued the expression of key differentiation markers normally induced by calcium and suppressed by E6/E7, such as IVL, TGM1, S100A8, S100A9 (Fig. 8B-C, S6A-B) [11]. These results demonstrate that NSD2 is a key mediator of the hr-HPV-mediated impairment of epithelial differentiation and that its E6/E7-induced overexpression abrogates the physiological epithelial differentiation process induced by extracellular CaCl_2_.

To corroborate the involvement of NSD2 into epithelial differentiation, we also overexpressed NSD2 in HKs (Fig. S6C). Coherently, NSD2 OE significantly upregulates ΔNp63α and downregulates a panel of epithelial differentiation markers, either at the mRNA and protein levels (Fig. 8D, G). Morphologically, it resulted in a notable reduction in cell size, consistent with impaired differentiation and increased proliferative potential (Fig. 8E). Moreover, upon differentiation-inducing CaCl_2_ treatment, NSD2-overexpressing keratinocytes retained an undifferentiated morphology (Fig. 8F) and showed both decreased IVL and increased ΔNp63α protein and mRNA levels, compared to the empty-treated control (Fig. 8G, S6D-F), confirming the NSD2 inhibitory effect on the epithelial differentiation process. Consistent with these findings, we observed that NSD2 overexpression in HKs also increases the percentage of ALDH + cells, supporting its role in regulating the stem-like phenotype (Fig. S6G).

To finally validate these findings in patients’ samples, we performed IHC on normal and HNSCC tissues. In normal epithelia, NSD2 and ΔNp63α are strongly expressed in the nuclei of the Ki67 positive and highly proliferative cells of the basal layer (Fig. 8H, S6H). Then, their expression progressively diminishes moving towards the upper and more differentiated layers of the epithelium, where, conversely, the expression of IVL and TGM1 differentiation markers, increase (Fig. 8H). Interestingly, a similar pattern is observed in HNSCC tissues. NSD2 and ΔNp63α are strongly expressed in tumoral regions, particularly in HPV+ samples, where their expression, as previously demonstrated (Fig. 2) [68], is sustained by the E6/E7 oncoviral proteins. The center of tumoral areas, especially in HPV- tumors, is characterized by progressively differentiating and keratinizing cells. Herein, we observed the decrease of NSD2 signal and increase of the expression of the differentiation markers IVL and TGM1 (Fig. 8H).

To further support these findings, we analyzed RNA-seq data of TCGA HNSCC dataset and found a significant inverse correlation between NSD2 expression and a broad panel of epithelial differentiation markers (Fig. 8I, S6I).

Altogether, our data identify for the first time NSD2 as a key negative regulator of epithelial cell differentiation. Moreover, we observed that its overexpression, particularly in HPV+ HNSCC, contributes to the poorly differentiated phenotype of these tumors. In conclusion, our results provide insight into its potential as a therapeutic target for HNSCC, especially for HPV+ HNSCC.

## Discussion

HPV+ and HPV− HNSCC subtypes differ markedly at molecular and clinicopathological levels [3, 4, 73]. Despite these differences, current treatment strategies do not distinguish between the subtypes, highlighting the urgent need for more targeted and tailored therapies [3, 4, 7].

Epigenetic alterations are considered one of the key “hallmarks” of cancer, influencing multiple oncogenic pathways and are emerging as promising targets for cancer treatment [17, 18]. Coherently with the known alterations induced by hr-HPV oncoproteins E6 and E7 on the host epigenetic landscape, HPV+ and HPV- HNSCC are characterized by significant differences in their epigenetic profiles [14–16]. Although DNA methylation has been extensively studied in this context, a comprehensive analysis of hPTMs remains lacking [16, 19, 74]. In this study, we conducted a first-of-its-kind comparative analysis of histone H3 and H4 hPTMs in HPV+ and HPV− HNSCC. Using a highly quantitative MS approach, we profiled hPTMs in primary oropharyngeal tumor samples and a panel of HNSCC cell lines. Despite substantial overall heterogeneity, we identified key subtype-specific hPTMs, with notable differences at H3K36 and H3K27 histone marks. H3K36me2 was consistently upregulated in HPV+ HNSCC samples, including both patient specimens and cell lines, compared to the HPV- ones. Notably, our data revealed a novel direct role for HPV oncoproteins in this regulation, demonstrating that E6 and E7 increase H3K36me2 levels. H3K36me2 has a well-established oncogenic role, and previous studies described that it is overexpressed in HNSCC samples compared to normal tissues [24, 61]. Overall, these findings suggest that H3K36me2 may play a key role in HNSCC tumorigenesis and may be differently regulated and functionally implicated depending on the HPV status of the tumor. Altered global enrichment levels of specific histone marks are emerging as promising new biomarkers for patient stratification and our findings fit well in this context [61]. Interestingly, the substantial heterogeneity observed in hPTMs enrichment across the analyzed samples, may reflect the overall heterogeneity previously described in both HNSCC tumors and cell lines [73]. Also, it could be informative to explore whether specific mutations or molecular alterations in certain cell lines or patients contribute to the observed variations in hPTM enrichment. Moreover, as previous studies have proposed, additional criteria for subclassifying HPV+ and HPV- HNSCC [75–80], our findings highlight the importance of investigating whether some subgroups display distinct epigenetic signatures, potentially contributing to the observed heterogeneity. Further investigations are thus needed to unveil these aspects and to determine the potential correlation of H3K36me2 with tumor aggressiveness, therapeutic responses, and other clinicopathological features.

Given that changes in hPTM levels often result from dysregulated histone modifiers, we performed RNA-seq analysis on keratinocytes overexpressing E6/E7. NSD2, a histone methyltransferase known to regulate H3K36 methylation, emerged as the most strongly upregulated epigenetic enzyme. This suggests that HPV increases H3K36me2 levels at least in part by upregulating NSD2. Importantly, this effect was specific to high-risk HPV strains (HPV16/18), further suggesting a link of NSD2 to HPV-mediated oncogenesis. To date, this is the first direct evidence of hr-HPV E6/E7–driven upregulation of NSD2 and H3K36me2, with the exception of a TCGA-based study [25], that reported only a positive correlation between E6/E7 and NSD2 in HNSCC patients.

Although the regulatory mechanisms were not explored in this study, we hypothesize that NSD2 may be induced by hypomethylation of its promoter [81]. The involvement of the EZH2-NSD2 axis is plausible. Indeed, E6/E7 also induce the expression of EZH2, which, in other models, has been shown to upregulate NSD2 by repressing the transcription of specific NSD2-targeting miRNAs [34, 82].

Consistent with this HPV-mediated regulation, both HNSCC cell lines and patient tissue samples exhibited higher NSD2 levels in HPV+ HNSCC compared to the HPV-, mirroring a similar pattern of H3K36me2. Beyond confirming these data, analysis of TCGA datasets and of patient samples, further revealed that NSD2 is overexpressed in both HPV+ and HPV- HNSCC compared to normal tissues. These findings suggest that, in addition to HPV-mediated mechanisms, alternative regulatory pathways contribute, albeit to a lesser extent, to NSD2 overexpression in the HPV-negative subtype.

Notably, our results highlight how both NSD2 and H3K36me2 may represent novel clinical biomarkers for HNSCC. Due to the promising role of liquid biopsies in early diagnosis of HNSCC, together with recent development of methods enabling the analysis of cf-RNA and cf-nucleosomes [83–85], it would be of great interest to investigate the potential of both NSD2 and H3K36me2 as early and/or prognostic biomarkers in plasma samples.

NSD2, a member of the NSD histone methyltransferase family, is aberrantly expressed in various cancers, including HNSCC. Being an epigenetic regulator, NSD2 is potentially druggable. Over the past five years, interest for this protein in cancer research, has increased significantly, with notable efforts focused on identifying new and effective inhibitors against NSD2 [65, 86]. This interest is further supported by the recent advancement of one NSD2-targeting compound into a phase I clinical trial (https://www.clinicaltrials.gov/study/NCT05651932).

While the oncogenic role of NSD2 is well-established, emerging evidence suggests that NSD2 functions are cell type– and context–dependent [27, 28]. Prior studies have mainly focused on NSD2 involvement in cell proliferation and EMT, without examining differences between HPV+ and HPV− subtypes [23, 24].

Our results confirmed that NSD2 promotes cell proliferation, EMT, and migration in both subtypes, reinforcing its role as an oncogene and a potential therapeutic target across HNSCC subtypes. Notably, through RNA-seq analysis of a panel of NSD2-silenced HNSCC cell lines, we identified distinct gene clusters regulated in a subtype-specific manner. One gene cluster, predominantly upregulated in HPV+ cells, was linked to keratinocyte and epithelial cell differentiation. We found that high level of NSD2 impair epithelial cell differentiation, also in presence of high CaCl_2_ concentrations, a known inducer of epithelial differentiation. Additionally, the levels of NSD2 were inversely correlated with the expression levels of differentiation markers in both HNSCC patient tissue samples and normal epithelia.

Our findings reveal a previously unrecognized role for NSD2 in epithelial differentiation and tissue homeostasis. This aligns with the known functions of NSD2 and H3K36me2 in regulating cell plasticity and maintaining cell identity. The stronger differentiation-inducing effect of the NSD2 silencing on HPV+ cell lines compared to the HPV-, could be due to the well-established E6/E7-mediated suppression of epithelial differentiation [10, 11]. Moreover, HPV+ HNSCC tend to be less differentiated and exhibit higher histological grades than HPV− tumors, suggesting that NSD2 inhibition might promote differentiation also in HPV− cases, albeit to a lesser extent [3, 4].

In this context, we also discovered that elevated NSD2 expression leads to increased levels of ΔNp63α—the predominant TP63 isoform in epithelial tissues and squamous cell carcinoma. ΔNp63α is a master regulator of cell differentiation, is essential for maintaining the self-renewal capacity and proliferative state of the basal cells of the epithelium and is considered a key oncogene in HNSCC. Like NSD2, it is upregulated by E6/E7 and is crucial for HPV-driven tumorigenesis [87]. Our data reveal for the first time that NSD2 acts as an upstream regulator of ΔNp63α. Both proteins are co-expressed in the basal layers of epithelial tissues and progressively lost in more differentiated, keratinized layers. This regulatory relationship has significant therapeutic implications, especially since targeting ΔNp63α is emerging as a promising strategy for squamous cell carcinoma treatment and, in this context, epigenetic therapies may offer an effective approach [69, 88]. The acquisition of stem-like properties and of self-renewal potential is a key process during cancer development. We found that NSD2 also affects ALDH cellular positivity and sphere forming capacity, suggesting a role in regulating stem-like features of the cells, consistently with previous observations from other models [22, 32]. Activation of cellular differentiation was predominantly observed in the tested HPV+ HNSCC cell lines, while in the HPV- subtype more heterogeneous and less consistent results were observed. As previously mentioned, this difference may reflect the distinct biological drivers of these subtypes: HPV+ HNSCC are mainly driven by hr-HPV oncoviral proteins, which are known to impair differentiation programs [10, 11], while HPV- HNSCC are characterized by marked inter-tumoral heterogeneity due to diverse mutational landscapes, which may differentially affect the differentiation pathways and their modulation upon NSD2 targeting. Nevertheless, we cannot exclude the possibility that NSD2 inhibition may also promote cellular differentiation also in some specific subtypes of HPV- HNSCC. Further studies are required to identify these responsive subgroups and define their molecular characteristics.

In this context, from a mechanistic point of view, NSD2 was reported to promote stem-like features in HNSCC cell lines by methylating histone H1 [26]. This indicates that NSD2 may affect cell plasticity through multiple mechanisms and regulatory networks that may include not only the redistribution and reshaping of H3K36me2 epigenomic profiles, but also the methylation of other unexplored histone targets, as it is for H1, or non-histone targets as PTEN and FANCM [89, 90]. Moreover, transcription factors that are in their turn regulated by NSD2, could be also implicated. This is the case of ΔNp63α, which works as a crucial epidermal pioneer factor, enhancing chromatin accessibility at epidermal enhancers, cooperating with specific transcription factors and epigenetic modulators during keratinocyte maturation [88]. Regarding H3K36me2, conversely to our observations, some studies showed that significant reductions in its levels, induced by H3K36M or NSD1 inactivating mutations (mainly described in HPV- tumors), downregulate differentiation genes and induce a differentiation blockade [75]. However, H3K36M is known to induce a dramatic loss of all H3K36 methylation states (me1, -me2 and -me3), while NSD2 knock-out or knock-down specifically affects H3K36me2 levels and reshapes both the genome-wide and gene-specific distribution of this mark. This, in turn, influences the genome-wide distribution of the repressive H3K27me3 and the activating H3K27ac histone modifications as well as DNA methylation profiles [91, 92]. Thus, we hypothesize that NSD2 affects the epigenome and transcriptional program of the cell in a specific and distinct manner compared to H3K36M, which may conceivably exert a more widespread effect. Moreover, NSD1 and NSD2 have been described as two structural and functional paralogues with non-redundant roles [75, 93]. Overall, we hypothesize that complex, interconnected and cell-specific networks are involved in NSD2-oncogenic function and, more specifically, in its mediated regulation of differentiation programs. Further investigation and epigenomic analyses are needed to clarify how H3K36M, NSD2 and NSD1 differently affect H3K36me2 deposition and redistribution, how they functionally interact with DNA methylation, and how H3K36me2 regulates cell lineage identity and differentiation in a context-dependent manner. Additionally, their potentially different effects on other methylation targets also warrants further explorations.

## Conclusions

In conclusion, we identified H3K36me2 has a potential novel biomarker for patient stratification and highlighted NSD2 as a key oncogene and promising therapeutic target in HNSCC. NSD2 and H3K36me2 are upregulated in response to high-risk HPV infection and play crucial roles in promoting oncogenesis and suppressing differentiation in HPV + tumors. The subtype-specific regulation of cellular differentiation in HPV+ HNSCC also suggests that combining NSD2 inhibitors with context-specific drugs could significantly improve patient outcomes and fits well with the promising and, in some cases well-assessed, efficacy of differentiation-inducing therapies [12, 13]. Additionally, given the NSD2 role in DNA damage regulation and that low ALDH1 and high Fibronectin levels have been shown to enhance sensitivity to radiotherapy [22, 94–96], further studies may unveil whether inhibiting NSD2 may sensitize cells to conventional chemo- and radiotherapies in HNSCC, as seen in different systems [22, 89], thus conceivably allowing personalized treatment strategies.

## Supplementary Information

Supplementary Material 1.

Supplementary Material 2.

Supplementary Material 3.

Supplementary Material 4.

Supplementary Material 5.

Supplementary Material 6.

Supplementary Material 7.

Supplementary Material 8.

Supplementary Material 9.

## Data Availability

All NGS data of this study (raw and processed) are deposited in the gene expressionomnibus (GEO) database under the accession number GSE313615. All the mass spectrometry data have been deposited to the ProteomeXchange Consortium (47) via the PRIDE partner repository with the dataset identifier PXD064152. All relevant data are within the paper and its Supporting Information files; further inquiries can be directed to the corresponding author.

## Abbreviations

AJCC: American Joint Commission on Cancer
ALDH: Aldehyde Dehydrogenase
CSCs: cancer stem cells
DEAB: 4-(Diethylamino)benzaldehyde
DEGs: Differentially expressed genes
ΔNp63α: Delta N p63 Alpha
EMT: Epithelial-to-Mesenchymal Transition
FDR: False Discovery Rate
FFPE: Formalin-Fixed Paraffin-Embedded
FLG: Filaggrin
FN1: Fibronectin
GO: Gene Ontology
GSEA: Gene Set Enrichment Analysis
HNC: Head and Neck Cancer
HNSCC: Head and Neck Squamous Cell Carcinoma
hPTMs: histone Post-Translational Modifications
HPV-: HPV-negative
HPV: Human Papillomavirus
HPV+: HPV-positive
hr-HPV: high-risk HPV
IEO: European Institute of Oncology
IHC: Immunohistochemistry
IVL: Involucrin
KD: Knock-down
lr-HPV: low-risk HPV
MMSET: multiple myeloma SET domain
MS: Mass Spectrometry
NSD2: nuclear receptor binding SET domain protein 2
OE: overexpression
OPSCC: oropharyngeal squamous cell carcinomas
RNA-seq: RNA-sequencing
RT-qPCR: Quantitative Reverse Transcription Polymerase Chain Reaction
S100A8: S100 Calcium Binding Protein A8
S100A9: S100 Calcium Binding Protein A9
SNAI2: Snail Family Transcriptional Repressor 2
SPRR2A: Small Proline Rich Protein 2 A
SPRR2E: Small Proline Rich Protein 2E
SPRR3: Small Proline Rich Protein 3
super-SILAC: Stable-Isotope Labeling using Amino acids in Cell culture
TGM1: Transglutaminase-1
VIM: Vimentin
WB: Western Blot
WHSC1: Wolf-Hirschhorn syndrome candidate 1

## References

[1] Bhat GR, Hyole RG, Li J. Head and neck cancer: current challenges and future perspectives. Adv Cancer Res. 2021;152:67–102. 34353444 10.1016/bs.acr.2021.05.002

[2] Bray F, Ferlay J, Soerjomataram I, Siegel RL, Torre LA, Jemal A. Global cancer statistics 2018: GLOBOCAN estimates of incidence and mortality worldwide for 36 cancers in 185 countries. CA Cancer J Clin. 2018;68(6):394–424. 30207593 10.3322/caac.21492

[3] Leemans CR, Snijders PJF, Brakenhoff RH. The molecular landscape of head and neck cancer. Nat Rev Cancer. 2018;18(5):269–82. 29497144 10.1038/nrc.2018.11

[4] Johnson DE, Burtness B, Leemans CR, Lui VWY, Bauman JE, Grandis JR. Head and neck squamous cell carcinoma. Nat Rev Dis Primers. 2020;6(1):92. 33243986 10.1038/s41572-020-00224-3 PMC7944998

[5] Boscolo-Rizzo P, Del Mistro A, Bussu F, Lupato V, Baboci L, Almadori G, et al. New insights into human papillomavirus-associated head and neck squamous cell carcinoma. Acta Otorhinolaryngol Ital. 2013;33(2):77–87. 23853396 PMC3665382

[6] Amin MB, Greene FL, Edge SB, Compton CC, Gershenwald JE, Brookland RK, et al. The eighth edition AJCC cancer staging manual: continuing to build a Bridge from a population-based to a more personalized approach to cancer staging. CA Cancer J Clin. 2017;67(2):93–9. 28094848 10.3322/caac.21388

[7] Cramer JD, Burtness B, Le QT, Ferris RL. The changing therapeutic landscape of head and neck cancer. Nat Rev Clin Oncol. 2019;16(11):669–83. 31189965 10.1038/s41571-019-0227-z

[8] Tommasino M. The human papillomavirus family and its role in carcinogenesis. Semin Cancer Biol. 2014;26:13–21. 24316445 10.1016/j.semcancer.2013.11.002

[9] Scarth JA, Patterson MR, Morgan EL, Macdonald A. The human papillomavirus oncoproteins: a review of the host pathways targeted on the road to transformation. J Gen Virol. 2021;102(3). 10.1099/jgv.0.001540. 10.1099/jgv.0.001540 PMC8148304 33427604

[10] Chong JS, Doorbar J. Modulation of epithelial homeostasis by HPV using Notch and Wnt. Tumour Virus Res. 2024;18:200297. 39542216 10.1016/j.tvr.2024.200297 PMC11617312

[11] White EA. Manipulation of epithelial differentiation by HPV oncoproteins. Viruses. 2019;11(4). 10.3390/v11040369. 10.3390/v11040369 PMC6549445 31013597

[12] Enane FO, Saunthararajah Y, Korc M. Differentiation therapy and the mechanisms that terminate cancer cell proliferation without harming normal cells. Cell Death Dis. 2018;9(9):912. 30190481 10.1038/s41419-018-0919-9 PMC6127320

[13] de The H. Differentiation therapy revisited. Nat Rev Cancer. 2018;18(2):117–27. 29192213 10.1038/nrc.2017.103

[14] Obanya DI, Wootton LM, Morgan EL. Advances in Understanding the mechanisms of the human papillomavirus oncoproteins. Biochem Soc Trans. 2025;53(3):565–77. 40380881 10.1042/BST20253041 PMC12224896

[15] Durzynska J, Lesniewicz K, Poreba E. Human papillomaviruses in epigenetic regulations. Mutat Res Rev Mutat Res. 2017;772:36–50. 28528689 10.1016/j.mrrev.2016.09.006

[16] Ghiani L, Chiocca S. High Risk-Human papillomavirus in HNSCC: present and future challenges for epigenetic therapies. Int J Mol Sci. 2022;23(7). 10.3390/ijms23073483. 10.3390/ijms23073483 PMC8998945 35408843

[17] Dawson MA, Kouzarides T. Cancer epigenetics: from mechanism to therapy. Cell. 2012;150(1):12–27. 22770212 10.1016/j.cell.2012.06.013

[18] Hillyar C, Rallis KS, Varghese J. Advances in epigenetic cancer therapeutics. Cureus. 2020;12(11):e11725. 33391954 10.7759/cureus.11725 PMC7772155

[19] Castilho RM, Squarize CH, Almeida LO. Epigenetic modifications and head and neck cancer: implications for tumor progression and resistance to therapy. Int J Mol Sci. 2017;18(7). 10.3390/ijms18071506. 10.3390/ijms18071506 PMC5535996 28704968

[20] Hudlebusch HR, Santoni-Rugiu E, Simon R, Ralfkiaer E, Rossing HH, Johansen JV, et al. The histone methyltransferase and putative oncoprotein MMSET is overexpressed in a large variety of human tumors. Clin Cancer Res. 2011;17(9):2919–33. 21385930 10.1158/1078-0432.CCR-10-1302

[21] Kuo AJ, Cheung P, Chen K, Zee BM, Kioi M, Lauring J, et al. NSD2 links dimethylation of histone H3 at lysine 36 to oncogenic programming. Mol Cell. 2011;44(4):609–20. 22099308 10.1016/j.molcel.2011.08.042 PMC3222870

[22] Chen R, Chen Y, Zhao W, Fang C, Zhou W, Yang X, et al. The role of methyltransferase NSD2 as a potential oncogene in human solid tumors. Onco Targets Ther. 2020;13:6837–46. 32764971 10.2147/OTT.S259873 PMC7367929

[23] Zhang L, Hu G. NSD2 activates the E2F transcription factor 1/Y-box binding protein 2 axis to promote the malignant development of oral squamous cell carcinoma. Arch Oral Biol. 2022;138:105412. 35364436 10.1016/j.archoralbio.2022.105412

[24] Saloura V, Cho HS, Kiyotani K, Alachkar H, Zuo Z, Nakakido M, et al. WHSC1 promotes oncogenesis through regulation of NIMA-related kinase-7 in squamous cell carcinoma of the head and neck. Mol Cancer Res. 2015;13(2):293–304. 25280969 10.1158/1541-7786.MCR-14-0292-T

[25] Gameiro SF, Ghasemi F, Zeng PYF, Mundi N, Howlett CJ, Plantinga P, et al. Low expression of NSD1, NSD2, and NSD3 define a subset of human papillomavirus-positive oral squamous carcinomas with unfavorable prognosis. Infect Agent Cancer. 2021;16(1):13. 33588906 10.1186/s13027-021-00347-6 PMC7885607

[26] Saloura V, Vougiouklakis T, Bao R, Kim S, Baek S, Zewde M, et al. WHSC1 monomethylates histone H1 and induces stem-cell like features in squamous cell carcinoma of the head and neck. Neoplasia. 2020;22(8):283–93. 32497898 10.1016/j.neo.2020.05.002 PMC7265065

[27] Pierro J, Saliba J, Narang S, Sethia G, Saint Fleur-Lominy S, Chowdhury A, et al. The NSD2 p.E1099K mutation is enriched at relapse and confers drug resistance in a cell Context-Dependent manner in pediatric acute lymphoblastic leukemia. Mol Cancer Res. 2020;18(8):1153–65. 32332049 10.1158/1541-7786.MCR-20-0092 PMC7415532

[28] Oyer JA, Huang X, Zheng Y, Shim J, Ezponda T, Carpenter Z, et al. Point mutation E1099K in MMSET/NSD2 enhances its methyltranferase activity and leads to altered global chromatin methylation in lymphoid malignancies. Leukemia. 2014;28(1):198–201. 23823660 10.1038/leu.2013.204 PMC3888226

[29] Yuan S, Natesan R, Sanchez-Rivera FJ, Li J, Bhanu NV, Yamazoe T, et al. Global regulation of the histone mark H3K36me2 underlies epithelial plasticity and metastatic progression. Cancer Discov. 2020;10(6):854–71. 32188706 10.1158/2159-8290.CD-19-1299 PMC7269857

[30] Park JW, Kang JY, Hahm JY, Kim HJ, Seo SB. Proteosomal degradation of NSD2 by BRCA1 promotes leukemia cell differentiation. Commun Biol. 2020;3(1):462. 32826945 10.1038/s42003-020-01186-8 PMC7443147

[31] Hoetker MS, Yagi M, Di Stefano B, Langerman J, Cristea S, Wong LP, et al. H3K36 methylation maintains cell identity by regulating opposing lineage programmes. Nat Cell Biol. 2023;25(8):1121–34. 37460697 10.1038/s41556-023-01191-z PMC10896483

[32] Hudlebusch HR, Skotte J, Santoni-Rugiu E, Zimling ZG, Lees MJ, Simon R, et al. MMSET is highly expressed and associated with aggressiveness in neuroblastoma. Cancer Res. 2011;71(12):4226–35. 21527557 10.1158/0008-5472.CAN-10-3810

[33] Ko EK, Anderson A, D’Souza C, Zou J, Huang S, Cho S, et al. Disruption of H3K36 methylation provokes cellular plasticity to drive aberrant glandular formation and squamous carcinogenesis. Dev Cell. 2024;59(2):187–98. e7. 38198888 10.1016/j.devcel.2023.12.007 PMC10872381

[34] Asangani IA, Ateeq B, Cao Q, Dodson L, Pandhi M, Kunju LP, et al. Characterization of the EZH2-MMSET histone methyltransferase regulatory axis in cancer. Mol Cell. 2013;49(1):80–93. 23159737 10.1016/j.molcel.2012.10.008 PMC3547524

[35] Lu C, Jain SU, Hoelper D, Bechet D, Molden RC, Ran L, et al. Histone H3K36 mutations promote sarcomagenesis through altered histone methylation landscape. Science. 2016;352(6287):844–9. 27174990 10.1126/science.aac7272 PMC4928577

[36] Citro S, Bellini A, Miccolo C, Ghiani L, Carey TE, Chiocca S. Synergistic antitumour activity of HDAC inhibitor SAHA and EGFR inhibitor gefitinib in head and neck cancer: a key role for DeltaNp63alpha. Br J Cancer. 2019;120(6):658–67. 30765872 10.1038/s41416-019-0394-9 PMC6461861

[37] Olthof NC, Huebbers CU, Kolligs J, Henfling M, Ramaekers FC, Cornet I, et al. Viral load, gene expression and mapping of viral integration sites in HPV16-associated HNSCC cell lines. Int J Cancer. 2015;136(5):E207–18. 25082736 10.1002/ijc.29112 PMC5370555

[38] Carey TE, Van Dyke DL, Worsham MJ, Bradford CR, Babu VR, Schwartz DR, et al. Characterization of human laryngeal primary and metastatic squamous cell carcinoma cell lines UM-SCC-17A and UM-SCC-17B. Cancer Res. 1989;49(21):6098–107. 2790823

[39] Mattoscio D, Casadio C, Miccolo C, Maffini F, Raimondi A, Tacchetti C, et al. Autophagy regulates UBC9 levels during viral-mediated tumorigenesis. PLoS Pathog. 2017;13(3):e1006262. 28253371 10.1371/journal.ppat.1006262 PMC5349695

[40] Medda A, Compagnoni M, Spini G, Citro S, Croci O, Campaner S, et al. c-MYC-dependent transcriptional Inhibition of autophagy is implicated in cisplatin sensitivity in HPV-positive head and neck cancer. Cell Death Dis. 2023;14(11):719. 37925449 10.1038/s41419-023-06248-3 PMC10625625

[41] Noberini R, Restellini C, Savoia EO, Bonaldi T. Enrichment of histones from patient samples for mass spectrometry-based analysis of post-translational modifications. Methods. 2020;184:19–28. 31605746 10.1016/j.ymeth.2019.10.001

[42] Noberini R, Bonaldi T. A Super-SILAC strategy for the accurate and multiplexed profiling of histone posttranslational modifications. Methods Enzymol. 2017;586:311–32. 28137569 10.1016/bs.mie.2016.09.036

[43] Noberini R, Longhi E, Bonaldi T. A Super-SILAC approach for profiling histone posttranslational modifications. Methods Mol Biol. 2023;2603:87–102. 36370272 10.1007/978-1-0716-2863-8_7

[44] Noberini R, Savoia EO, Brandini S, Greco F, Marra F, Bertalot G, et al. Spatial epi-proteomics enabled by histone post-translational modification analysis from low-abundance clinical samples. Clin Epigenetics. 2021;13(1):145. 34315505 10.1186/s13148-021-01120-7 PMC8317427

[45] Yuan ZF, Sidoli S, Marchione DM, Simithy J, Janssen KA, Szurgot MR, et al. EpiProfile 2.0: A computational platform for processing Epi-Proteomics mass spectrometry data. J Proteome Res. 2018;17(7):2533–41. 29790754 10.1021/acs.jproteome.8b00133 PMC6387837

[46] Ritchie ME, Phipson B, Wu D, Hu Y, Law CW, Shi W, et al. Limma powers differential expression analyses for RNA-sequencing and microarray studies. Nucleic Acids Res. 2015;43(7):e47. 25605792 10.1093/nar/gkv007 PMC4402510

[47] Perez-Riverol Y, Bai J, Bandla C, Garcia-Seisdedos D, Hewapathirana S, Kamatchinathan S, et al. The PRIDE database resources in 2022: a hub for mass spectrometry-based proteomics evidences. Nucleic Acids Res. 2022;50(D1):D543–52. 34723319 10.1093/nar/gkab1038 PMC8728295

[48] Bianchi V, Ceol A, Ogier AG, de Pretis S, Galeota E, Kishore K, et al. Integrated systems for NGS data management and analysis: open issues and available solutions. Front Genet. 2016;7:75. 27200084 10.3389/fgene.2016.00075 PMC4858535

[49] Kim D, Pertea G, Trapnell C, Pimentel H, Kelley R, Salzberg SL. TopHat2: accurate alignment of transcriptomes in the presence of insertions, deletions and gene fusions. Genome Biol. 2013;14(4):R36. 23618408 10.1186/gb-2013-14-4-r36 PMC4053844

[50] Love MI, Huber W, Anders S. Moderated Estimation of fold change and dispersion for RNA-seq data with DESeq2. Genome Biol. 2014;15(12):550. 25516281 10.1186/s13059-014-0550-8 PMC4302049

[51] Subramanian A, Tamayo P, Mootha VK, Mukherjee S, Ebert BL, Gillette MA, et al. Gene set enrichment analysis: a knowledge-based approach for interpreting genome-wide expression profiles. Proc Natl Acad Sci U S A. 2005;102(43):15545–50. 16199517 10.1073/pnas.0506580102 PMC1239896

[52] Bankhead P, Loughrey MB, Fernández JA, Dombrowski Y, Mcart DG, Dunne PD et al. QuPath: open source software for digital pathology image analysis. Sci Rep. 2017;7. 10.1038/s41598-017-17204-5. 10.1038/s41598-017-17204-5 PMC5715110 29203879

[53] Schmidt U, Weigert M, Broaddus C, Myers G. Cell Detection with Star-Convex Polygons. Pt Ii. 2018;11071:265–73. Medical Image Computing and Computer Assisted Intervention - Micca. 2018.

[54] Weigert M, Schmidt U, Haase R, Sugawara K, Myers G. Star-convex Polyhedra for 3D Object Detection and Segmentation in Microscopy. 2020 Ieee Winter Conference on Applications of Computer Vision (Wacv). 2020:3655-62. 10.1109/WACV45572.2020.9093435.

[55] Weigert M, Schmidt U. Ieee Isbi. Nuclei Instance Segmentation and Classification in Histopathology Images with Stardist. 2022 Ieee International Symposium on Biomedical Imaging Challenges (2022). 2022. 10.1109/ISBIC56247.2022.9854534.

[56] Stringer C, Wang T, Michaelos M, Pachitariu M. Cellpose: a generalist algorithm for cellular segmentation. Nat Methods. 2021;18(1):100–. 33318659 10.1038/s41592-020-01018-x

[57] Cerami E, Gao J, Dogrusoz U, Gross BE, Sumer SO, Aksoy BA, et al. The cBio cancer genomics portal: an open platform for exploring multidimensional cancer genomics data. Cancer Discov. 2012;2(5):401–4. 22588877 10.1158/2159-8290.CD-12-0095 PMC3956037

[58] Gao J, Aksoy BA, Dogrusoz U, Dresdner G, Gross B, Sumer SO, et al. Integrative analysis of complex cancer genomics and clinical profiles using the cBioPortal. Sci Signal. 2013;6(269):pl1. 23550210 10.1126/scisignal.2004088 PMC4160307

[59] Fonseca TC, Jural LA, Maranon-Vasquez GA, Magno MB, Roza A, Ferreira D, et al. Global prevalence of human papillomavirus-related oral and oropharyngeal squamous cell carcinomas: a systematic review and meta-analysis. Clin Oral Investig. 2023;28(1):62. 38158517 10.1007/s00784-023-05425-0

[60] Noberini R, Uggetti A, Pruneri G, Minucci S, Bonaldi T. Pathology Tissue-quantitative mass spectrometry analysis to profile histone Post-translational modification patterns in patient samples. Mol Cell Proteom. 2016;15(3):866–77. 10.1074/mcp.M115.054510 PMC4813706 26463340

[61] Noberini R, Restellini C, Savoia EO, Raimondi F, Ghiani L, Jodice MG et al. Profiling of epigenetic features in clinical samples reveals novel widespread changes in cancer. Cancers (Basel). 2019;11(5). 10.3390/cancers11050723. 10.3390/cancers11050723 PMC6562406 31137727

[62] Doorbar J, Egawa N, Griffin H, Kranjec C, Murakami I. Human papillomavirus molecular biology and disease association. Rev Med Virol. 2015;25(1):2–23. 25752814 10.1002/rmv.1822 PMC5024016

[63] Martinez-Garcia E, Popovic R, Min DJ, Sweet SM, Thomas PM, Zamdborg L, et al. The MMSET histone Methyl transferase switches global histone Methylation and alters gene expression in t(4;14) multiple myeloma cells. Blood. 2011;117(1):211–20. 20974671 10.1182/blood-2010-07-298349 PMC3037745

[64] He G, Ke Y, Yuan J, Zhang B, Dai L, Liu J, et al. NSD2-mediated H3K36me2 exacerbates osteoporosis via activation of hoxa2 in bone marrow mesenchymal stem cells. Cell Signal. 2024;121:111294. 38996954 10.1016/j.cellsig.2024.111294

[65] He L, Cao Y, Sun L. NSD family proteins: rising stars as therapeutic targets. Cell Insight. 2024;3(2):100151. 38371593 10.1016/j.cellin.2024.100151 PMC10869250

[66] Soares E, Zhou H. Master regulatory role of p63 in epidermal development and disease. Cell Mol Life Sci. 2018;75(7):1179–90. 29103147 10.1007/s00018-017-2701-z PMC5843667

[67] Sniezek JC, Matheny KE, Westfall MD, Pietenpol JA. Dominant negative p63 isoform expression in head and neck squamous cell carcinoma. Laryngoscope. 2004;114(12):2063–72. 15564824 10.1097/01.mlg.0000149437.35855.4b

[68] Citro S, Bellini A, Medda A, Sabatini ME, Tagliabue M, Chu F, et al. Human papilloma virus increases DeltaNp63alpha expression in head and neck squamous cell carcinoma. Front Cell Infect Microbiol. 2020;10:143. 32322564 10.3389/fcimb.2020.00143 PMC7156594

[69] Fisher ML, Balinth S, Mills AA. DeltaNp63alpha in cancer: importance and therapeutic opportunities. Trends Cell Biol. 2023;33(4):280–92. 36115734 10.1016/j.tcb.2022.08.003 PMC10011024

[70] Zhou C, Sun B. The prognostic role of the cancer stem cell marker aldehyde dehydrogenase 1 in head and neck squamous cell carcinomas: a meta-analysis. Oral Oncol. 2014;50(12):1144–8. 25264224 10.1016/j.oraloncology.2014.08.018

[71] Mahanty S, Dakappa SS, Shariff R, Patel S, Swamy MM, Majumdar A, et al. Keratinocyte differentiation promotes ER stress-dependent lysosome biogenesis. Cell Death Dis. 2019;10(4):269. 30890691 10.1038/s41419-019-1478-4 PMC6425001

[72] Hennings H, Holbrook KA. Calcium regulation of cell-cell contact and differentiation of epidermal cells in culture. An ultrastructural study. Exp Cell Res. 1983;143(1):127–42. 6186504 10.1016/0014-4827(83)90115-5

[73] Cheng H, Yang X, Si H, Saleh AD, Xiao W, Coupar J, et al. Genomic and transcriptomic characterization links cell lines with aggressive head and neck cancers. Cell Rep. 2018;25(5):1332–e455. 30380422 10.1016/j.celrep.2018.10.007 PMC6280671

[74] Degli Esposti D, Sklias A, Lima SC, Beghelli-de la Forest Divonne S, Cahais V, Fernandez-Jimenez N, et al. Unique DNA methylation signature in HPV-positive head and neck squamous cell carcinomas. Genome Med. 2017;9(1):33. 28381277 10.1186/s13073-017-0419-z PMC5382363

[75] Papillon-Cavanagh S, Lu C, Gayden T, Mikael LG, Bechet D, Karamboulas C, et al. Impaired H3K36 methylation defines a subset of head and neck squamous cell carcinomas. Nat Genet. 2017;49(2):180–5. 28067913 10.1038/ng.3757 PMC5549104

[76] Walter V, Yin X, Wilkerson MD, Cabanski CR, Zhao N, Du Y, et al. Molecular subtypes in head and neck cancer exhibit distinct patterns of chromosomal gain and loss of canonical cancer genes. PLoS ONE. 2013;8(2):e56823. 23451093 10.1371/journal.pone.0056823 PMC3579892

[77] Diao P, Dai Y, Wang A, Bu X, Wang Z, Li J, et al. Integrative multiomics analyses identify molecular subtypes of head and neck squamous cell carcinoma with distinct therapeutic vulnerabilities. Cancer Res. 2024;84(18):3101–17. 38959352 10.1158/0008-5472.CAN-23-3594

[78] Zhang Y, Koneva LA, Virani S, Arthur AE, Virani A, Hall PB, et al. Subtypes of HPV-Positive head and neck cancers are associated with HPV Characteristics, copy number Alterations, PIK3CA Mutation, and pathway signatures. Clin Cancer Res. 2016;22(18):4735–45. 27091409 10.1158/1078-0432.CCR-16-0323 PMC5026546

[79] Qin T, Li S, Henry LE, Liu S, Sartor MA. Molecular tumor subtypes of HPV-Positive head and neck cancers: biological characteristics and implications for clinical outcomes. Cancers (Basel). 2021;13(11). 10.3390/cancers13112721. 10.3390/cancers13112721 PMC8198180 34072836

[80] Carrero I, Liu HC, Sikora AG, Milosavljevic A. Histoepigenetic analysis of HPV- and tobacco-associated head and neck cancer identifies both subtype-specific and common therapeutic targets despite divergent microenvironments. Oncogene. 2019;38(19):3551–68. 30655605 10.1038/s41388-018-0659-4 PMC6756123

[81] Yin Z, Sun Y, Ge S, Sun J. Epigenetic activation of WHSC1 functions as an oncogene and is associated with poor prognosis in cervical cancer. Oncol Rep. 2017;37(4):2286–94. 28260054 10.3892/or.2017.5463

[82] Hyland PL, McDade SS, McCloskey R, Dickson GJ, Arthur K, McCance DJ, et al. Evidence for alteration of EZH2, BMI1, and KDM6A and epigenetic reprogramming in human papillomavirus type 16 E6/E7-expressing keratinocytes. J Virol. 2011;85(21):10999–1006. 21865393 10.1128/JVI.00160-11 PMC3194988

[83] Galati L, Tagliabue M, Gheit T, De Berardinis R, Maffini F, McKay-Chopin S, et al. HPV biomarkers in oral and Blood-Derived body fluids in head and neck cancer patients. J Med Virol. 2025;97(3):e70278. 40052199 10.1002/jmv.70278 PMC11886502

[84] Meng R, Gao Y, Peng Z, Chen H, Li Y, Liu Y et al. Development and validation of a small extracellular vesicle-derived RNA signature for early diagnosis of lung adenocarcinoma and prognosis in advanced stages. Mol Cancer. 2025. 10.1186/s12943-025-02524-2. 10.1186/s12943-025-02524-2 PMC12781256 41331809

[85] Van den Ackerveken P, Lobbens A, Pamart D, Kotronoulas A, Rommelaere G, Eccleston M, et al. Epigenetic profiles of elevated cell free Circulating H3.1 nucleosomes as potential biomarkers for non-Hodgkin lymphoma. Sci Rep. 2023;13(1):16335. 37770512 10.1038/s41598-023-43520-0 PMC10539380

[86] Zuo Z, Ran J, Zhou Y, Chen M. Targeting nuclear receptor-binding SET domain protein 2 (NSD2) for cancer therapy: challenges and emerging strategies. Bioorg Chem. 2025;168:109344. 41386065 10.1016/j.bioorg.2025.109344

[87] Loljung L, Coates PJ, Nekulova M, Laurell G, Wahlgren M, Wilms T, et al. High expression of p63 is correlated to poor prognosis in squamous cell carcinoma of the tongue. J Oral Pathol Med. 2014;43(1):14–9. 23607508 10.1111/jop.12074

[88] Yi M, Tan Y, Wang L, Cai J, Li X, Zeng Z, et al. TP63 links chromatin remodeling and enhancer reprogramming to epidermal differentiation and squamous cell carcinoma development. Cell Mol Life Sci. 2020;77(21):4325–46. 32447427 10.1007/s00018-020-03539-2 PMC7588389

[89] Zhang J, Lee YR, Dang F, Gan W, Menon AV, Katon JM, et al. PTEN methylation by NSD2 controls cellular sensitivity to DNA damage. Cancer Discov. 2019;9(9):1306–23. 31217297 10.1158/2159-8290.CD-18-0083 PMC6726527

[90] Weirich S, Kusevic D, Schnee P, Reiter J, Pleiss J, Jeltsch A. Discovery of NSD2 non-histone substrates and design of a super-substrate. Commun Biol. 2024;7(1):707. 38851815 10.1038/s42003-024-06395-z PMC11162472

[91] Lhoumaud P, Badri S, Rodriguez-Hernaez J, Sakellaropoulos T, Sethia G, Kloetgen A, et al. NSD2 overexpression drives clustered chromatin and transcriptional changes in a subset of insulated domains. Nat Commun. 2019;10(1):4843. 31649247 10.1038/s41467-019-12811-4 PMC6813313

[92] Popovic R, Martinez-Garcia E, Giannopoulou EG, Zhang Q, Zhang Q, Ezponda T, et al. Histone methyltransferase MMSET/NSD2 alters EZH2 binding and reprograms the myeloma epigenome through global and focal changes in H3K36 and H3K27 methylation. PLoS Genet. 2014;10(9):e1004566. 25188243 10.1371/journal.pgen.1004566 PMC4154646

[93] Wagner RT, Hlady RA, Pan X, Wang L, Kim S, Zhao X, et al. SETD2 loss-of-function uniquely sensitizes cells to epigenetic targeting of NSD1-directed H3K36 methylation. Genome Biol. 2025;26(1):22. 39910618 10.1186/s13059-025-03483-z PMC11800516

[94] Shah MY, Martinez-Garcia E, Phillip JM, Chambliss AB, Popovic R, Ezponda T, et al. MMSET/WHSC1 enhances DNA damage repair leading to an increase in resistance to chemotherapeutic agents. Oncogene. 2016;35(45):5905–15. 27109101 10.1038/onc.2016.116 PMC6071667

[95] Kurth I, Hein L, Mabert K, Peitzsch C, Koi L, Cojoc M, et al. Cancer stem cell related markers of radioresistance in head and neck squamous cell carcinoma. Oncotarget. 2015;6(33):34494–509. 26460734 10.18632/oncotarget.5417 PMC4741468

[96] Jerhammar F, Ceder R, Garvin S, Grenman R, Grafstrom RC, Roberg K. Fibronectin 1 is a potential biomarker for radioresistance in head and neck squamous cell carcinoma. Cancer Biol Ther. 2010;10(12):1244–51. 20930522 10.4161/cbt.10.12.13432

