# Supplementary material for: NSD2 upregulation is driven by high-risk HPV E6/E7 and disrupts epithelial differentiation in HPV-associated head and neck cancer
Source: J Exp Clin Cancer Res. 2026 Jan 8;45:40. doi: 10.1186/s13046-025-03631-0 (PMC12882316; doi:10.1186/s13046-025-03631-0)
Supplement: Supplementary file 1 — Supplementary Material 1. [file 13046_2025_3631_MOESM1_ESM.docx]

Supplementary Tables

Table 1: siRNA sequences used in this study

|  | *siRNA sequences* |
| --- | --- |
| siLuc | 5’- CGUACGGGGAAUACUUCGA -3’ sense  5’- UCGAAGUAUUCCCCGUACG -3’ antisense |
| siE6/E7 | 5’-CUUCGGUUGUGCGUACAAAGC -3’ sense  5’- GCUUUGUACGCACAACCGAAG-3’ antisense |

Table 2: shRNA sequences used in this study

|  | *shNSD2 oligos for cloning* |
| --- | --- |
| ShNSD2_A | FW: CCGGCGGAAAGCCAAGTTCACCTTTCTCGAGAAAGGTGAACTTGGCTTTCC  GTTTTTG  RV: AATTCAAAAACGGAAAGCCAAGTTCACCTTTCTCGAGAAAGGTGAACTTGGC  TTTCCG |
| ShNSD2_B | FW: CCGGATCTTACTTCCCGGGTGTTTACTCGAGTAAACACCCGGGAAGTAAGAT  TTTTTG  RV: AATTCAAAAAATCTTACTTCCCGGGTGTTTACTCGAGTAAACACCCGGGAAGT  AAGAT |

Table 3: RT-qPCR Primers used in this study

|  | *RT-qPCR Primers* |
| --- | --- |
| RPLP0 | FW: TTCATTGTGGGAGCAGAC  RV: CAGCAGTTTCTCCAGAGC |
| HPV6-E6 | FW: ATGCACTGACCACAGCAGAG  RV: GCGGTTTGTGACACAGGTAG |
| HPV6-E7 | FW: ATGAGGTGGACGAAGTGGAC  RV: CGCAGATGGGACACACTATG |
| HPV10-E6 | FW: GGCATTGTACGACGCCTAA  RV: GGTCTCCTCTTCCACACCTTC |
| HPV10-E7 | FW: GAACCAGCACAACAAGCGTA  RV: TTATATCTGCGTGGCTGCAC |
| HPV16-E6 | FW: ATGTTTCAGGACCCACAGGA  RV: CAGCTGGGTTTCTCTACGTGTT |
| HPV16-E7 | FW: CAGAGGAGGAGGATGAAATAGATGG  RV: CACAACCGAAGCGTAGAGTCACAC |
| HPV18-E6 | FW: CTATAGAGGCCAGTGCCATTCG  RV: TTATACTTGTGTTTCTCTGCGTCG |
| HPV18-E7 | FW: GCGACTCAGAGGAAGAAAACGATG  RV: ACACCACGGACACACAAAGGACAG |
| NSD2 | FW: TTGGGAGAAATGGCAGAATC  RV: TCTGCCGTCTTTTGAGGAGT |
| VIM | FW: TTCTCAGCATCACGATGACC  RV: GCAGAAAGGCACTTGAAAGC |
| N-CAD | FW: GGACCGAGAATCACCAAATG  RV: AACACTTGAGGGGCATTGTC |
| FN-1 | FW: ACAACACCGAGGTGACTGAGAC  RV: GGACACAACGATGCTTCCTGAG |
| SNAI2 | FW: ACTGGACACACATACAGTGATTAT  RV: GAATGGAGCAGCGGTAGTC |
| ΔNp63α | FW: GGAAAACAATGCCCAGACTC  RV: GTGGAATACGTCCAGGTGGC |
| IVL | FW: TGCCTCAGCCTTACTGTGAG  RV: TCATTTGCTCCTGATGGGTA |
| TGM1 | FW: ATCCTCATGGTCCACGTACACA  RV: CCCCCGCAATGAGTCTACA |
| FLG | FW: GGGCACTGAAAGGCAAAAAG  RV: CACCATAATCATAATCTGCACTACCA |
| S100A8 | FW: GGGCATCAGTTGACCGAGC  RV: GTAACTCAGCTACTCTTTGTGGCTT |
| S100A9 | FW: CACCCAGACACCCTGAAC  RV: GCCCTCGTCACCCTCGTG |
| SPRR2A | FW: CAGCTTCAGAATTCATCAGGACCAA  RV: TGGGCAGATTACTGGCTAAGGA |
| SPRR2E | FW: CTCTGTCCCTGGGAACCAT  RV: CAGGTGTTAGAAGCCCATGC |
| SPRR3 | FW: AGCAGGTCCAGCATCCTTTGA  RV: CTCCTTGGTTGTGGGAACAAAT |

Table 4: FFPE Patient Tissue Samples analyzed by Mass Spectrometry

|  |  | |  | |
| --- | --- | --- | --- | --- |
|  |  |  |  |  |
|  | **OPC** | | |  |
|  | **HPV- (n=9)** | **HPV+ (n=19)** | |  |
| **Sex** |  |  | |  |
| F | 2 | 3 | |  |
| M | 7 | 16 | |  |
| **Histology** |  |  | |  |
| scc | 8 | 7 | |  |
| scc-basaloid | 1 | 12 | |  |
| **Histological Grade** |  |  | |  |
| 1 | - | - | |  |
| 2 | 3 | - | |  |
| 3 | 4 | 15 | |  |
| Missing information | 2 | 4 | |  |
| **pT VIII edition** |  |  | |  |
| 0 | - | 1 | |  |
| 1 | 1 | 5 | |  |
| 2 | 2 | 11 | |  |
| 3 | 3 | - | |  |
| 4 | 3 | 1 | |  |
| Missing information | - | 1 | |  |
| **pN VIII edition** |  |  | |  |
| 0 | 3 | 4 | |  |
| 1 | - | 11 | |  |
| 2 | 3 | 3 | |  |
| 3 | 1 | - | |  |
| x | 1 | - | |  |
| Missing information | 1 | 1 | |  |
| **pSTAGE VIII edition** |  |  | |  |
| 1 | 2 | 11 | |  |
| 2 | - | 3 | |  |
| 3 | 1 | - | |  |
| 4 | 3 | - | |  |
| Missing information | 3 | 5 | |  |
