# Supplementary material for: NSD2 upregulation is driven by high-risk HPV E6/E7 and disrupts epithelial differentiation in HPV-associated head and neck cancer
Source: J Exp Clin Cancer Res. 2026 Jan 8;45:40. doi: 10.1186/s13046-025-03631-0 (PMC12882316; doi:10.1186/s13046-025-03631-0)
Supplement: Supplementary file 3 — Supplementary Material 3. [file 13046_2025_3631_MOESM3_ESM.docx]

**Supplementary Figure Legends**

**Supplementary Figure S1**

**Controls of E6/E7-overexpressing HKs and siE6E7-transfected HNSCC cell lines. A)** Western Blot of of the same HKs transduced with HPV16 E6/E7 oncoviral proteins used for super-SILAC Mass Spectrometry Analysis in Fig. 1C. The figure shows the downregulation of p53 levels upon E6 overexpression. P53 was used as a surrogate marker of E6 overexpression. β-actin was used as a housekeeping control. **B)** Western Blot of HKs transduced with HPV16 E6/E7 oncoviral proteins and showing the H3K36me2 levels. H3 tot was used as a housekeeping control **C)** Gene Set Enrichment Analysis (GSEA) of RNA-seq data from HKs transduced with E6 and E7. Representative Enrichment plots of regulated genes in Human Primary Keratinocytes transduced with E6 and E7 are shown. Analysis has been performed through the GSEA software. **D)** Total mRNA was extracted from 5 HPV+ HNSCC cell lines transiently transfected with siLuc or siE6E7 sequences. The histograms show the HPV16- E6/ and E7 mRNA expression levels analyzed by RT-qPCR and normalized on the RPLP0 housekeeping genes. Values are represented as fold changes on the scrambled control and expressed as mean ± SD. **E)** Total mRNA was extracted from HKs overexpressing the E6/E7 genes of two low-risk (HPV-6, HPV-10) and two high-risk (HPV-16, HPV18) HPV genotypes. E6/E7 mRNA expression levels were analyzed by RT-qPCR and normalized on the RPLP0 housekeeping genes. Values are represented as fold changes on the scrambled control and expressed as mean ± SD.

**Supplementary Figure S2. NSD2 silencing (shNSD2_B) reduces cell proliferation and migration in HPV+ and HPV- HNSCC cell lines.** 4 HPV- and 4 HPV+ HNSCC cell lines were transduced with shNSD2 (_B) and scrambled control. **A)** Western Blots showing the NSD2 and H3K36me2 protein levels HPV- and HPV+ HNSCC cell lines upon NSD2 silencing. Vinculin and H4 total were respectively used as loading control. **B)** Viability of 2 HPV- (in blue) and 2 HPV+ (in red) shNSD2-transduced HNSCC cell lines was assessed using CellTiter-Glo^®^ Luminescent Cell Viability Assay and expressed as relative viability to the time point 0 (day 0) (means ± SD). Statistical analyses were performed using unpaired t-test*.* **C)** Colony Formation Assay performed on 2 HPV- (in blue) and 2 HPV+ (in red) NSD2-silenced (shNSD2_B) HNSCC cell lines. Representative images are reported for each condition and the respective histograms on the right, show the quantified number of colonies/well. Results are the average of at least two replicates. Unpaired t-test.  **D)** Western Blots showing the Vimentin protein levels in 2 HPV- and 2 HPV+ HNSCC cell lines, upon shNSD2_b transduction. Vinculin was used as a loading control. **E)** Phase contrast representative pictures of wound healing assays performed on an HPV- (UM-SCC-4) and an HPV+ (93-VU147T) HNSCC cell line transduced with shNSD2_b or scrambled control. Images were acquired at time 0 and 24hrs. Graphs on the right represent the percentage of wound closure and expressed as mean ± SD. For each cell line values represent the average of at least three independent replicates normalized relative to scrambled group average. Unpaired t-test**, p ≤ 0,05; ***, p ≤ 0,001.*

**Supplementary Figure S3. RNA-seq analysis on HPV+ and HPV- HNSCC cell lines upon shNSD2 silencing.** Differential gene expression analysis was performed comparing the shNSD2_A and scr conditions separately in HPV- and in HPV+ HNSCC cell lines. **A)** RNA-seq data shown in Fig.7A were used to generate a new heatmap showing the log2FC of the same genes of figure 7A for each single cell line. **B)** Box-Plot showing, for each cell line, the log2FC of genes of cluster 9. In blue are represented the HPV- HNSCC cell lines, in red the HPV+ ones. Mann-Withney test. **C)** Venn diagrams showing the number of downregulated and upregulated DEGs in HPV- and HPV+ subtypes upon shNSD2 stable transduction. Upon NSD2 silencing, 215 DEGs (117 up; 98 down) have been identified in the HPV+ subgroup and 415 (173 up; 242 down) in the HPV- one. 30 downregulated genes are in common between HPV+ and HPV- subtypes (left) while the shared upregulated genes were 35 (right). Thresholds used: FDR ≤ 0,05 and |Log2FC| > 0,5. Diagrams have been generated through the Venny 2.1 software **D-E)** Gene Set Enrichment Analysis of DEGs of HPV- and HPV+ HNSCC cell lines, upon NSD2 silencing**.** Representative Enrichment plots of positively regulated genes in HPV- HNSCC cell lines **(D)** and in HPV+ cell lines **(E)** upon shNSD2 stable transduction are shown. Analysis have been performed through the GSEA software.

**Supplementary Figure S4. NSD2 silencing stem cell-like properties in HPV+ HNSCC cell line smore consistently than in the HPV- ones.** **A)** Total mRNA was extracted from 3 HPV- (UM-SCC-6, UM-SCC-18, UM-SCC-19). The histograms shows the mRNA levels of a panel of epithelial cell differentiation markers, analyzed by RT-qPCR and normalized on the housekeeping gene RPLP0. Values are expressed as means ± SD. **B)** ALDH+ cells were detected and quantified through the ALDEFLUOR assay in the UM-SCC-6 and UM-SCC-19 HPV- HNSCC cell lines (in blue) and in the 93-VU147-T HPV+ HNSCC cell lines (in red), upon NSD2-silencing, and acquired by FACS analysis. Treatment with DEAB inhibitor was used as negative control. The histogram shows the fold change over the scr control of the detected percentage of ALDH+ cells, in at least two independent experiments, ±SD. One-sample t-test. **C)** Sphere formation assay was performed in in the UM-SCC-6 and UM-SCC-19 HPV- HNSCC cell lines and in the 93-VU147-T HPV+ HNSCC cell lines, upon NSD2-silencing. Images were acquired 15-20 days post-plating according to the cell line; spheres >70 µm in diameter were counted, except for 93-VU147-T, where spheres >50 µm in diameter were counted. The histogram shows the mean of two independent experiments ±SD. Unpaired t-test.

**Supplementary Figure S5. Immunofluorescence showing how NSD2 silencing restores morphological changes induced by CaCl_2_ treatment, in E6/E7 overexpressing keratinocytes.** Representative confocal images of HKs transduced with E6/E7-shNSD2 and their respective Empty-scr controls. After selection, cells were treated for 3 days with 1,2mM CaCl2. Phalloidin (red) was used to visualize cell size and morphology, nuclei were stained with DAPI (blue) and NSD2 was detected with Alexa 488 (green).

**Supplementary Figure S6. NSD2 arrest epithelial cell differentiation and negatively correlates with differentiation markers in HNSCC patients’ samples. A)** HKs were transduced with HPV16-E6/E7 encoding vector, with shNSD2 or scrambled control, and treated with 1,2 mM CaCl_2_. mRNA expression levels of NSD2, E6 and E7 were analyzed by RT-qPCR and normalized on RPLP0. Values of two technical replicates are expressed as means ±SD. **B)** Optic densitometric analysis of Immunoblot in figure 7B. Histogram showing values obtained quantifying the signal of IVL bands normalized on GAPDH. **C)** Total mRNA was extracted from HKs overexpressing NSD2. Histogram showing NSD2 mRNA levels analyzed by RT-qPCR and normalized on RPLP0. Values of at least three independent experiments are represented as fold changes on the empty vector control (±SD). One sample t-test. **D-F)** Total mRNA was extracted from HKs overexpressing NSD2 and treated with 1,2 mM CaCl_2_. **D)** Graph showing the NSD2 mRNA levels analyzed by RT-qPCR and normalized on RPLP0. Values of at least three independent experiments are represented as fold changes on the empty vector control and expressed as mean ±SD. Unpaired t-test. **E-F)** ΔNp63α and IVL mRNA expression levels were analyzed by RT-qPCR and normalized on RPLP0. Values of at least three independent experiments are shown as fold changes on the empty vector non-treated control and expressed as means ±SD. Unpaired t-test. **G)** ALDH+ cells in HKs overexpressing NSD2 were detected and quantified using the ALDEFLUOR assay and acquired by FACS analysis. Treatment with DEAB inhibitor was used as negative control. The histogram shows data obtained from two independent experiments. Values are expressed as mean ±SD. Paired t-test. **H)** Magnification of the IHC images in figure 8H, showing the levels of NSD2, ΔNp63α and Ki67. **I)** RNA-seq data from TCGA Dataset were downloaded and analyzed through the SRPlot software. Graphs show the correlation between the NSD2 and S100A8, S100A9, SPRR1A, SPRR2A, SPRR2E mRNA expression levels. Calculated Spearman Correlation were respectively of -0,39, -0,32, -0,31, -0,29, -0,33 (p < 0,0001). ***, p ≤ 0,01; ****, p ≤ 0,0001*
