# Supplementary figures and images for: NSD2 upregulation is driven by high-risk HPV E6/E7 and disrupts epithelial differentiation in HPV-associated head and neck cancer
Source: J Exp Clin Cancer Res. 2026 Jan 8;45:40. doi: 10.1186/s13046-025-03631-0 (PMC12882316; doi:10.1186/s13046-025-03631-0)

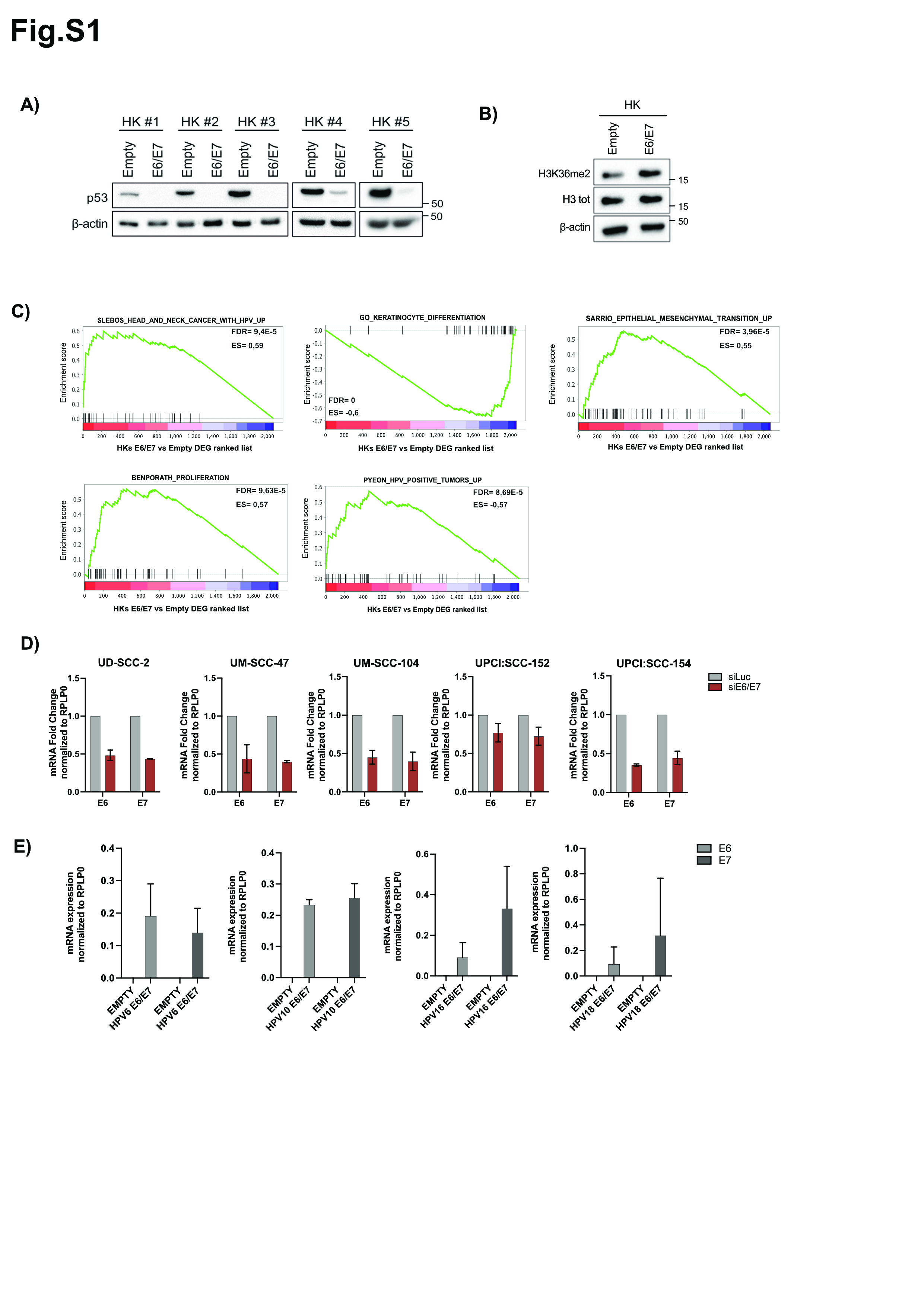

Supplement: Supplementary file 4 — Supplementary Material 4. [file 13046_2025_3631_MOESM4_ESM.tiff]

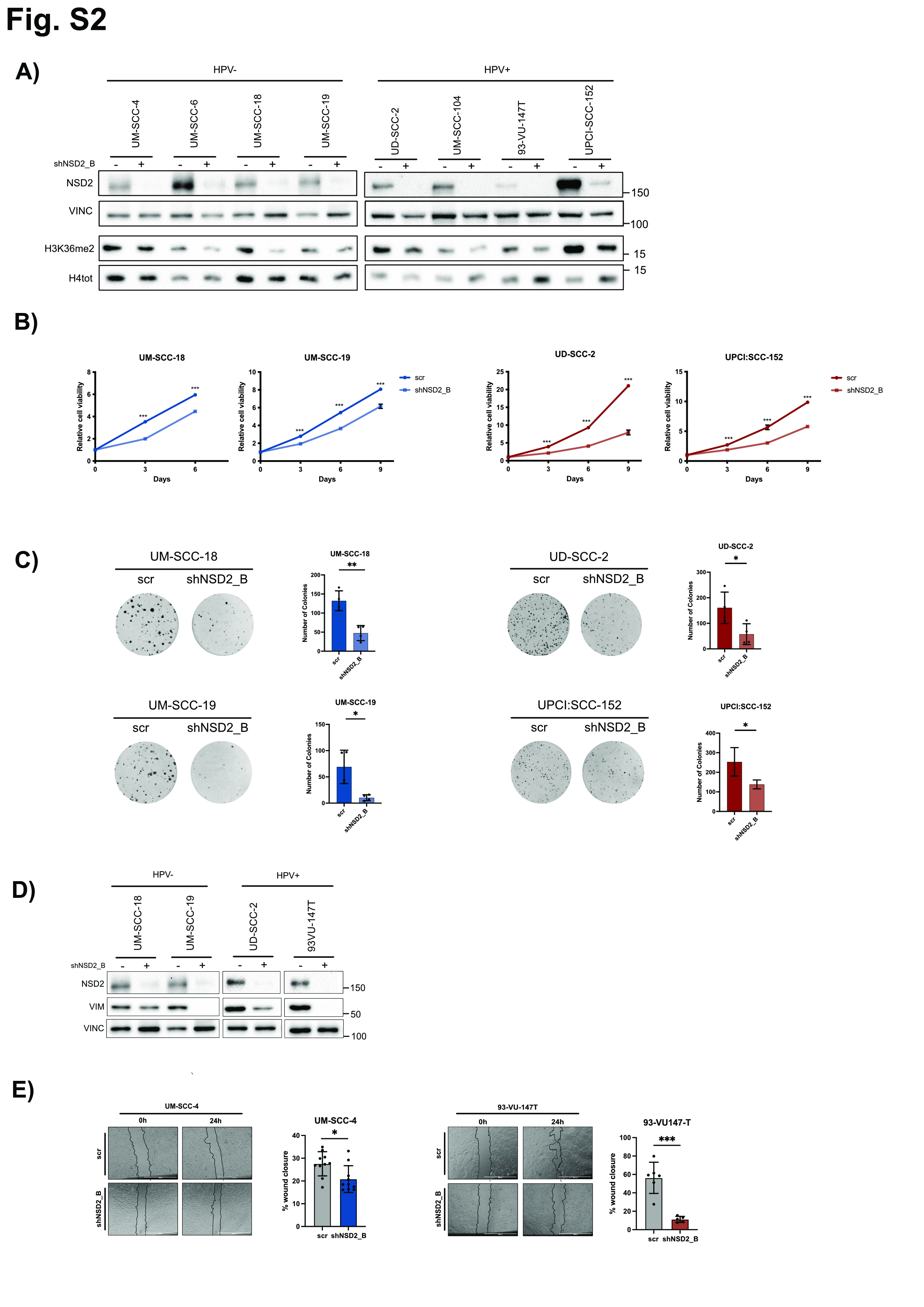

Supplement: Supplementary file 5 — Supplementary Material 5. [file 13046_2025_3631_MOESM5_ESM.tiff]

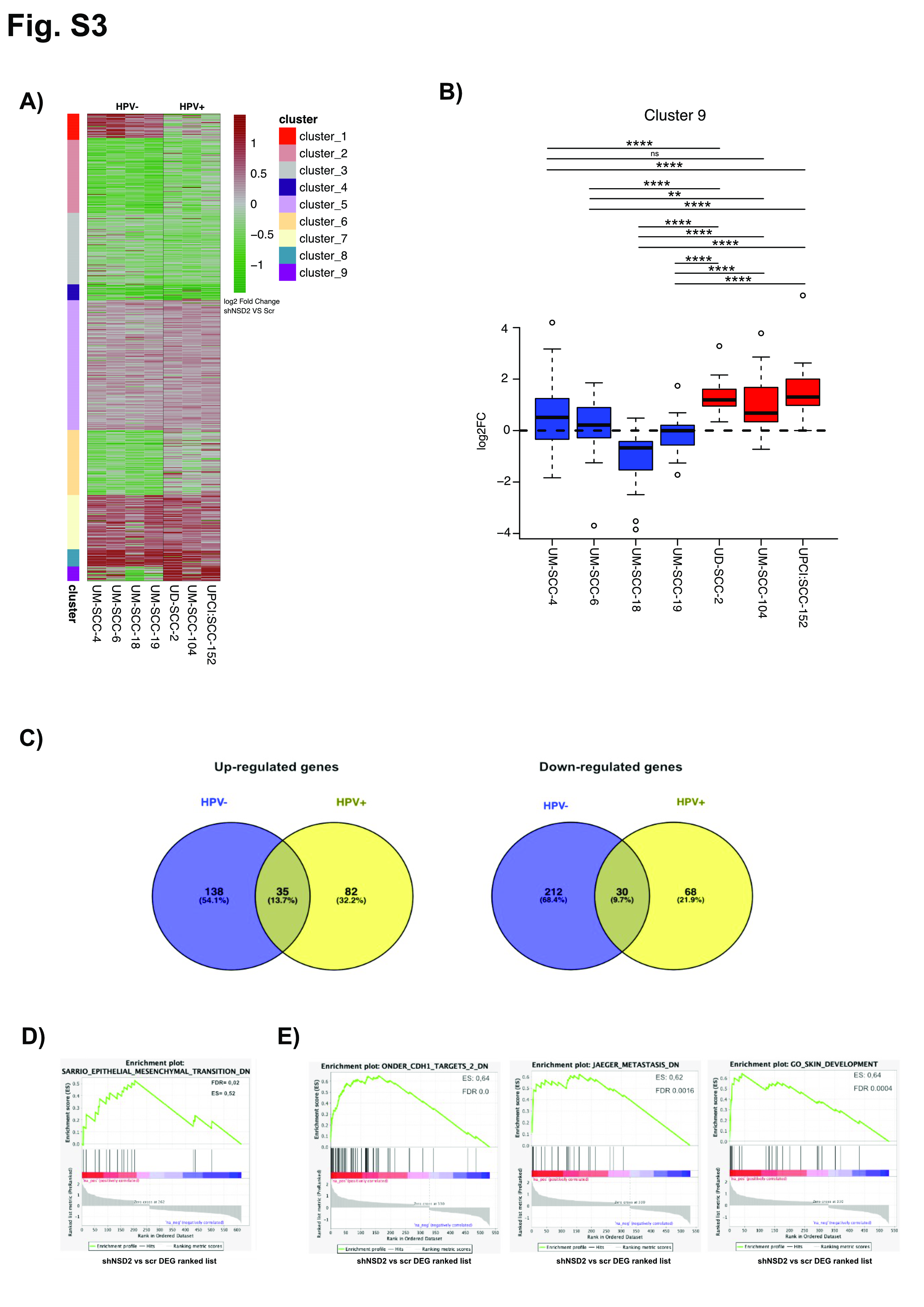

Supplement: Supplementary file 6 — Supplementary Material 6. [file 13046_2025_3631_MOESM6_ESM.tiff]

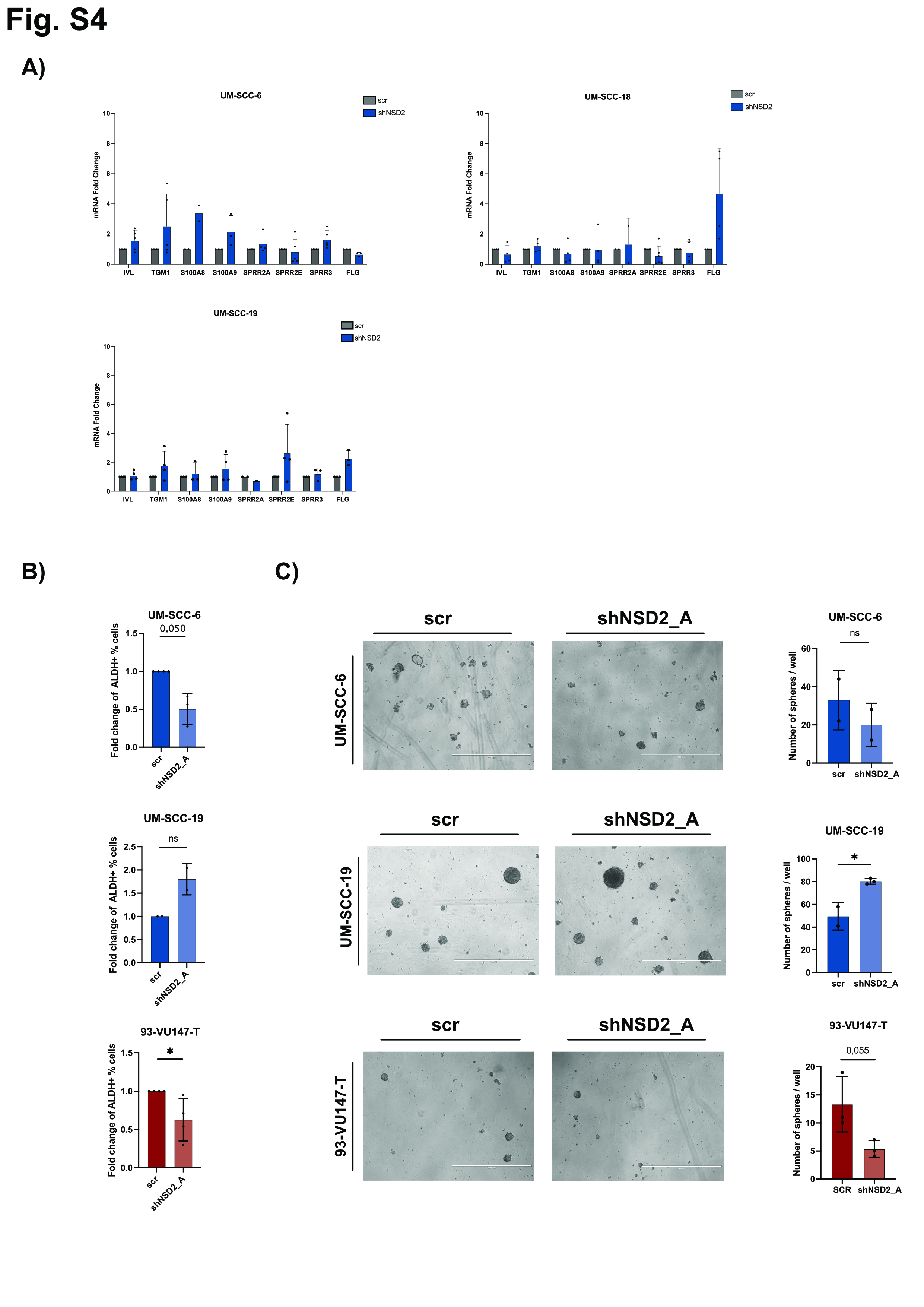

Supplement: Supplementary file 7 — Supplementary Material 7. [file 13046_2025_3631_MOESM7_ESM.tiff]

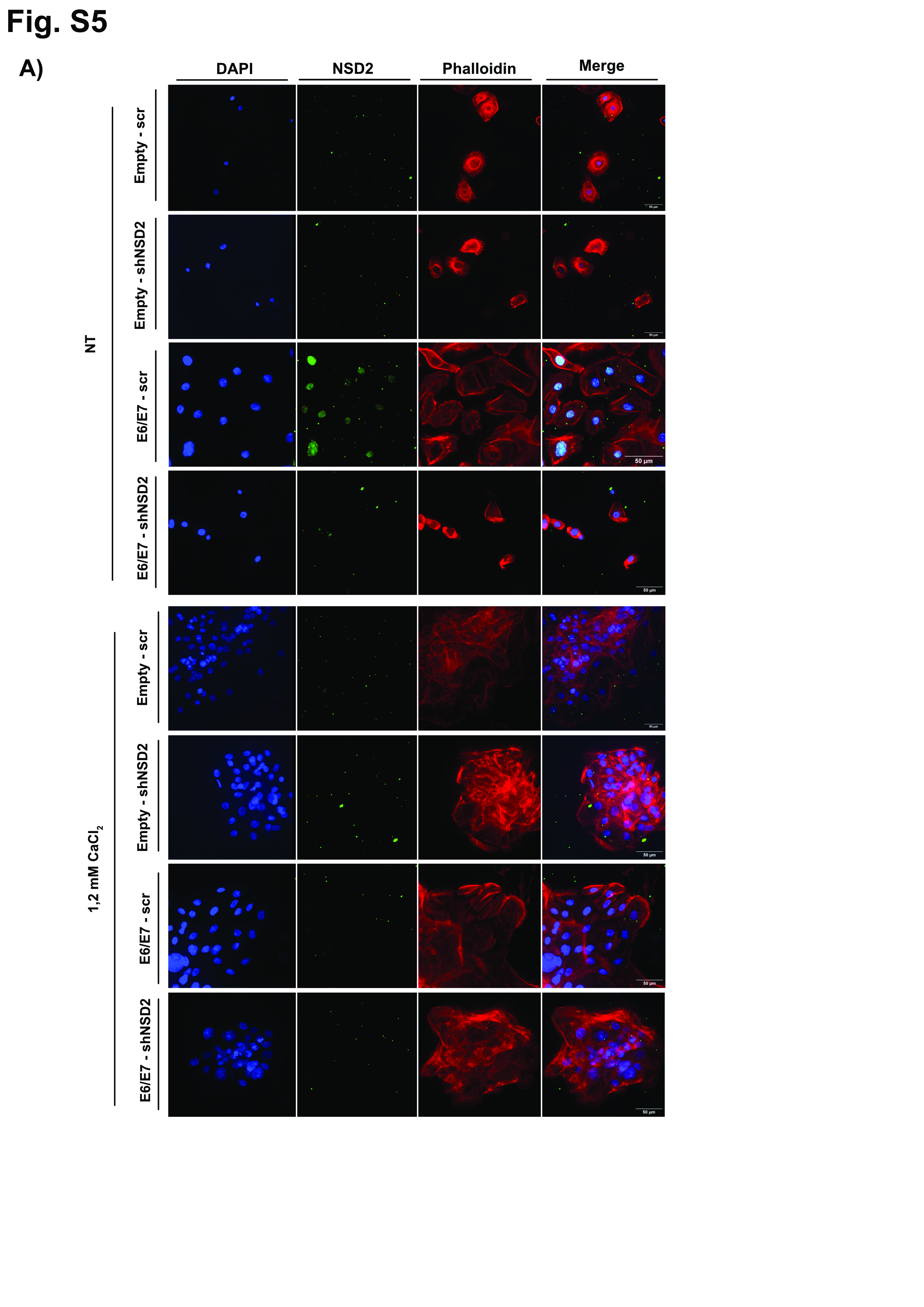

Supplement: Supplementary file 8 — Supplementary Material 8. [file 13046_2025_3631_MOESM8_ESM.tiff]

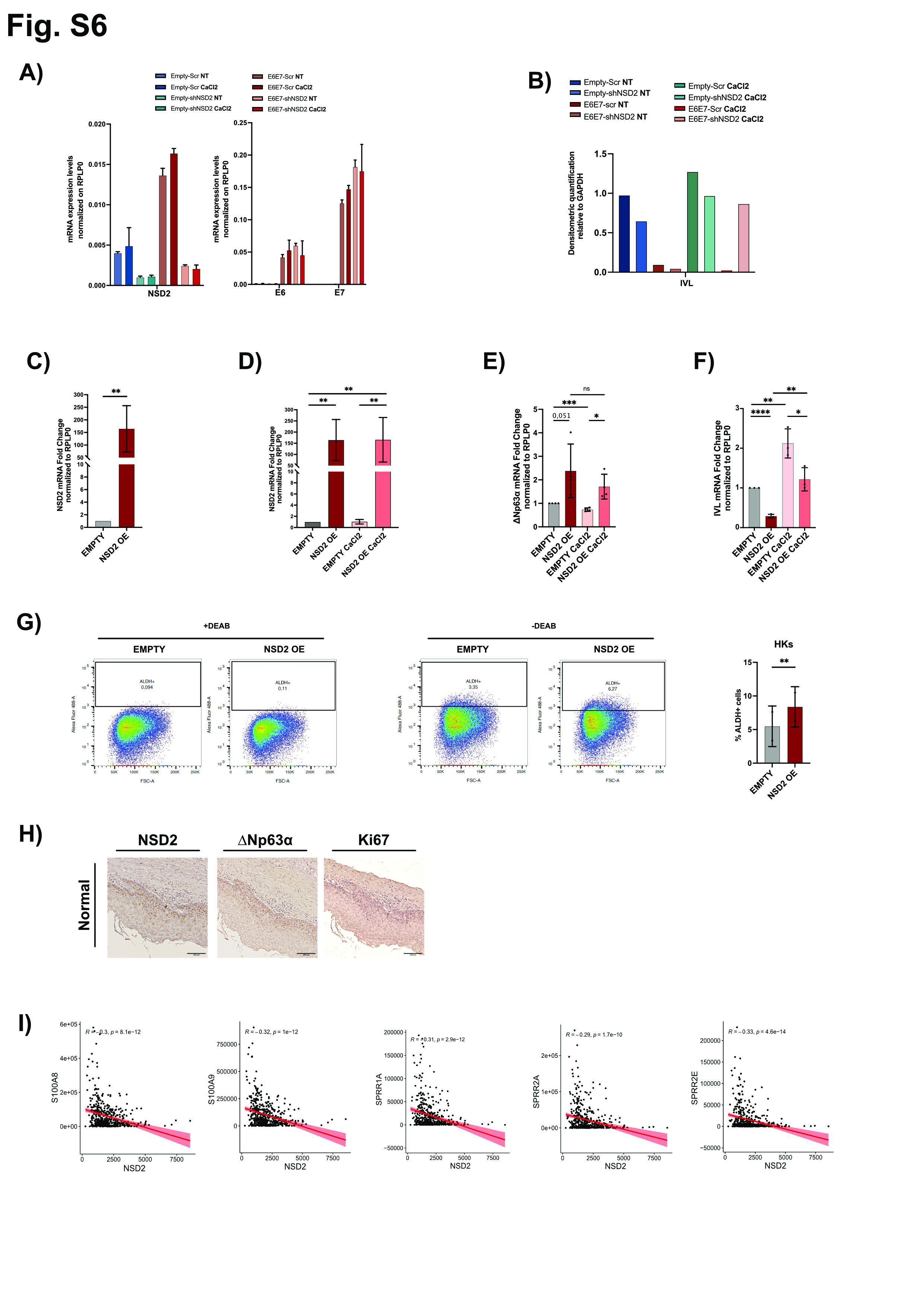

Supplement: Supplementary file 9 — Supplementary Material 9. [file 13046_2025_3631_MOESM9_ESM.tiff]
